# TLR4 regulates RORγt^+^ regulatory T-cell responses and susceptibility to colon inflammation through interaction with *Akkermansia muciniphila*

**DOI:** 10.1186/s40168-022-01296-x

**Published:** 2022-06-27

**Authors:** Yaojiang Liu, Min Yang, Li Tang, Fengchao Wang, Shengjie Huang, Shuang Liu, Yuanyuan Lei, Sumin Wang, Zhuo Xie, Wei Wang, Xiaoyan Zhao, Bo Tang, Shiming Yang

**Affiliations:** 1 Department of Gastroenterology, The Second Affiliated Hospital of Third Military Medical University, 400037 Chongqing, China; 2 Department of Gastroenterology, The Second Affiliated Hospital of Chongqing Medical University, Chongqing, 400010 China; 3 State Key Laboratory of Trauma, Burns and Combined Injury, Institute of Combined Injury, College of Preventive Medicine, Third Military Medical University, Chongqing, 400037 China

## Abstract

**Background:**

Well-balanced interactions between gut microbiota and the immune system are essential to prevent chronic intestinal inflammation, as observed in inflammatory bowel diseases (IBD). Toll-like receptor 4 (TLR4) functions as a sensor mediating the crosstalk between the intestinal commensal microbiome and host immunity, but the influence of TLR4 on the shaping of intestinal microbiota and immune responses during colon inflammation remains poorly characterized. We investigated whether the different susceptibilities to colitis between wild-type (WT) and TLR4^−/−^ mice were gut microbiota-dependent and aimed to identify the potential immunity modulation mechanism.

**Methods:**

We performed antibiotic depletion of the microbiota, cohousing experiments, and faecal microbiota transplantation (FMT) in WT and TLR4^−/−^ mice to assess the influence of TLR4 on intestinal microbial ecology. 16S rRNA sequencing was performed to dissect microbial discrepancies, and dysbiosis-associated immune perturbation was investigated by flow cytometry. *Akkermansia muciniphila* (*A. muciniphila*)-mediated immune modulation was confirmed through the T-cell transfer colitis model and bone marrow chimaera construction.

**Results:**

TLR4^−/−^ mice experienced enhanced susceptibility to DSS-induced colitis. 16S rRNA sequencing showed notable discrepancy in the gut microbiota between WT and TLR4^−/−^ mice. In particular, *A. muciniphila* contributed most to distinguishing the two groups. The T-cell transfer colitis model and bone marrow transplantation (BMT) consistently demonstrated that *A. muciniphila* ameliorated colitis by upregulating RORγt^+^ Treg cell-mediated immune responses. Mucosal biopsies from human manifested parallel outcomes with colon tissue from WT mice, as evidenced by the positive correlation between TLR4 expression and intestinal *A. muciniphila* colonization during homeostasis.

**Conclusions:**

Our results demonstrate a novel protective role of TLR4 against intestinal inflammation, wherein it can modulate *A. muciniphila*-associated immune responses. These findings provide a new perspective on host-commensal symbiosis, which may be beneficial for developing potential therapeutic strategies.

Video abstract.

**Graphical Abstract:**

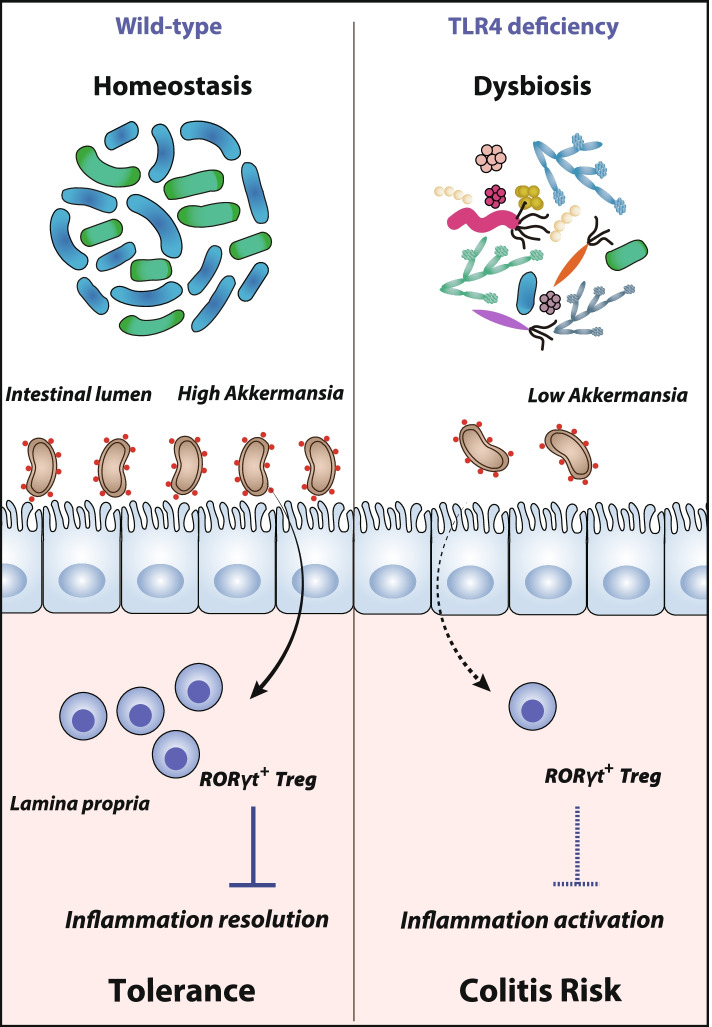

**Supplementary Information:**

The online version contains supplementary material available at 10.1186/s40168-022-01296-x.

## Introduction

Inflammatory bowel disease (IBD) encompasses two phenotypes, Crohn’s disease (CD) and ulcerative colitis (UC), which are characterized by chronic relapsing-remitting inflammatory disorder of the gastrointestinal tract [1, 2]. Although the exact aetiology remains unclear, it has long been recognized that the pathogenesis of IBD consists of a combination of genetic susceptibility, environmental exposure, gut microbiota disturbances, and immune system dysfunctions [3–5]. Notably, microbial dysbiosis, defined as a decrease in gut microbiome diversity owing to a shift in the balance between commensal and potentially pathogenic microorganisms, contributes to the occurrence of IBD [6–8]. Emerging evidence indicates that excessively activated immune responses, especially toll-like receptor (TLR)-dependent immune dysfunctions mediated through perturbations in the intestinal microbiome, play key roles in the etiopathogenesis of IBD [9–11].

Toll-like receptor 4 (TLR4), an essential member of the pattern-recognition receptor (PRR) family, functions as a key sensor of intestinal microbiota alterations and specifically recognizes pathogen-associated molecular patterns (PAMPs) and damage-associated molecular patterns (DAMPs) in the intestine [12, 13]. Increased expression of TLR4 is observed in epithelial and lamina propria cells of patients with IBD [14, 15]. Previous studies have demonstrated that TLR4 activation results in the transcription of inflammatory and immunoregulatory genes, and subsequent downstream signalling pathway cascades participate in the progression of IBD [16]. A large amount of evidence supports a negative proinflammatory role of the TLR4 signalling pathway in IBD. Interestingly, genetic mutations and dysregulations of TLRs (including TLR4) are associated with a markedly enhanced predisposition and susceptibility to IBD in animal models, indicating that TLR4 is required for the intestinal response to epithelial injury and commensal microflora recognition [17, 18]. TLR4 thus has dual roles in IBD; on the one hand, it can amplify inappropriate immune responses that ultimately cause chronic inflammation; on the other hand, it is necessary for maintaining tolerance and eliminating pathogenic microorganisms during steady-state conditions [19]. The impact of TLR4 on the aetiology of IBD is multidimensional and multifactorial, involving interactions among genetics, gut microbiota, and immune responses.

Prior studies have demonstrated that TLR4-deficient mice develop severe DSS-induced colitis, which is linked to impaired intestinal barrier function and changes in the inflammatory cytokine profile [18, 20]. Although TLR4 plays a decisive role in maintaining immune tolerance and gut homeostasis, its role in shaping colonic bacterial ecology and microbiota-associated immunity has not been investigated in depth. In this study, we investigated the impact of TLR4 on the shaping of intestinal microbiota and host immunity during colon inflammation. Here, we show that intestinal microbiota dysbiosis caused by the loss of TLR4 gives rise to imbalanced immune responses and an enhanced predisposition to colitis in mice. The reduced abundance of *Akkermansia muciniphila* (*A. muciniphila*) and the decreased frequency of suppressive RORγt^+^ Treg cells in TLR4^−/−^ mice contribute to the enhanced susceptibility to colon inflammation. These findings provide a new perspective on host-commensal symbiosis, which may be beneficial for the development of potential therapeutic strategies to alleviate IBD.

## Results

### Exacerbated colitis in TLR4^−/−^ mice depends on the gut microbiota

**Fig. 1:**
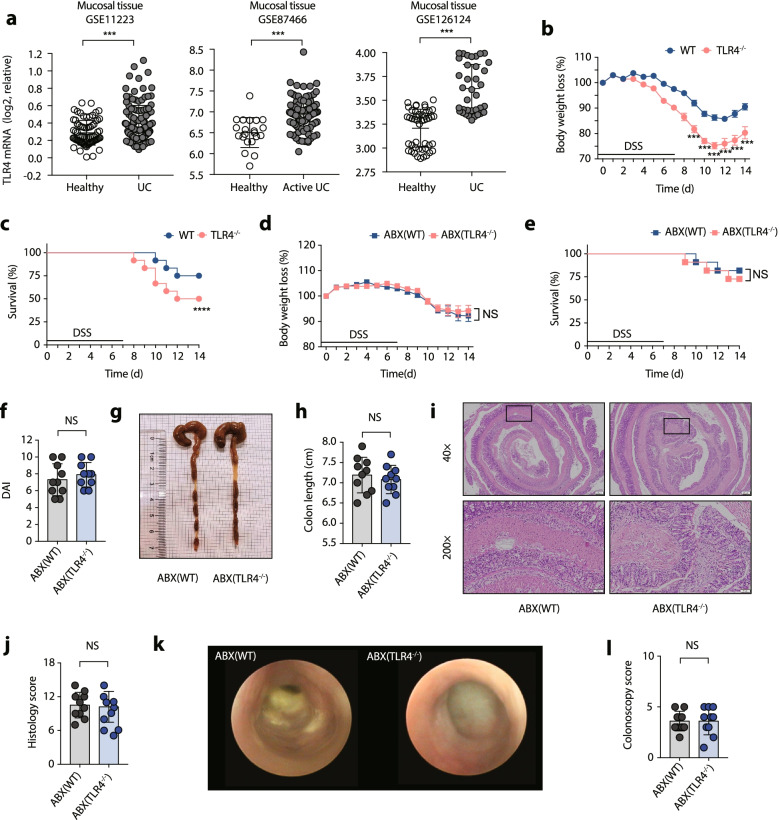
Gut microbiota distinguishes the colitis severity in WT mice from that in TLR4^−/−^ mice. **a** TLR4 expression is upregulated in tissue samples from UC patients by NCBI GEO database. (GSE11223: healthy sample, *n* = 73, UC patients, *n* = 128; GSE87466: healthy sample, *n* = 21, UC patients, *n* = 87; GSE126124: healthy sample, *n* = 47, UC patients, *n* = 36). **b** Body weight change. **c** Survival. **d** Body weight change. **e** Survival. **f** Disease activity index (DAI) score. **g** Representative pictures of colon gross appearance. **h** Colon length. **i** Representative microscopic pictures of H&E staining (40× and 200× magnification). **j** Histology score. **k** Representative colonoscopy images. (l) Colonoscopy score. (b-l) *n* = 10 mice per group, mean values ± SEM are presented, and *p*-values were calculated using unpaired *T*-test, *p < 0.05, **p < 0.01, ***p < 0.001. Data are pooled from three independent experiments with *n* = 10 mice per group

TLR4 gene expression was upregulated in the intestinal epithelia of patients with UC, indicating that TLR4 might be a participant in UC development (Fig. 1a). We observed that TLR4^−/−^ mice developed severe colitis, as evidenced by increased weight loss (Fig. 1b), decreased survival rate (Fig. 1c), a higher DAI score (Fig. S1a), and a shortened colon length (Fig. S1 b and c). Pronounced colon inflammation in TLR4^−/−^ mice was also obvious based on haematoxylin and eosin (H&E) staining of the histology score (Fig. S1 d and e) and endoscopic evaluations of the colonoscopy score (Fig. S1 f and g).

To assess the potential effect of the gut microbiota on the enhanced susceptibility of TLR4^−/−^ mice to colitis, WT and TLR4^−/−^ mice were gavaged with antibiotic cocktails for gut microbiota depletion [ABX(WT) vs. ABX(TLR4^−/−^)] before 1.5% DSS administration. In contrast to conventionally raised mice, ABX(WT) mice and ABX(TLR4^−/−^) mice showed indistinguishable body weight loss (Fig. 1d), mortality (Fig. 1e), DAI score (Fig. 1f), colon length (Fig. 1g and h), histology score (Fig. 1i and j), and colonoscopy score (Fig. 1k and l) following DSS treatment, indicating a role of gut microbiota in the severe colitis observed in TLR4^−/−^ mice.

### The gut microbiota differs between WT and TLR4^−/−^ mice

**Fig. 2:**
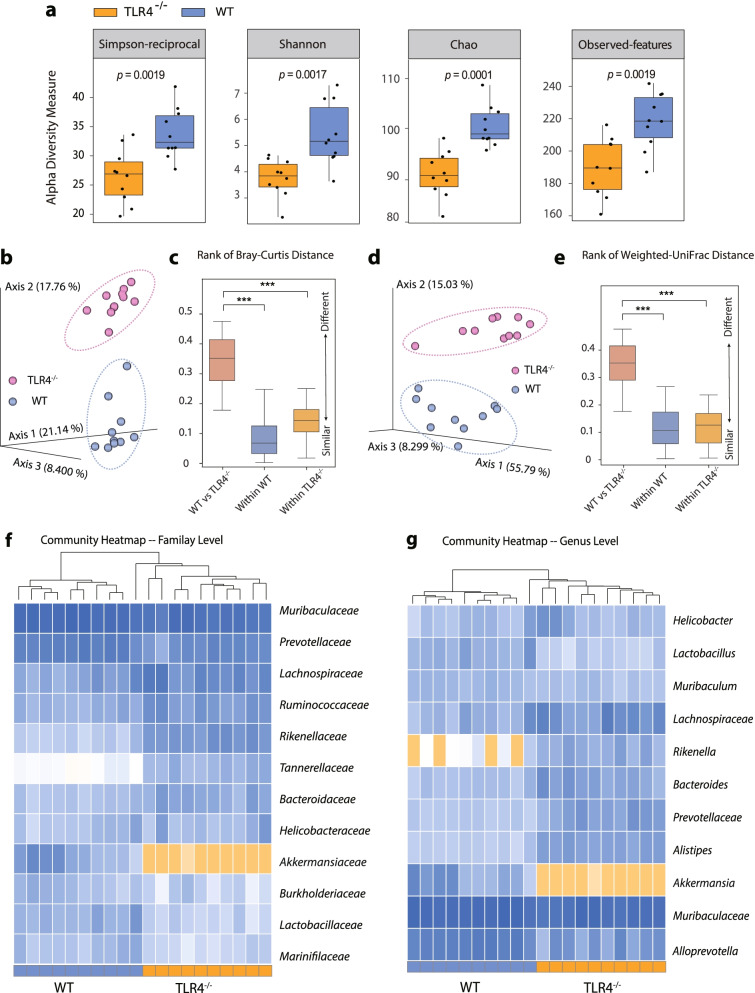
Loss of TLR4 promotes a dysbiotic and communicable microbiome. **a** Alpha diversity boxplot (based on Simpson reciprocal, Shannon, Chao, and observed features index). **b** Principal coordinate analysis (PCoA) using Bray-Curtis metric distances of beta diversity. **c** Quantification of dissimilarity values based on Bray-Curtis metric distances. **d** PCoA using weighted-UniFrac distances of beta diversity. **e** Quantification of dissimilarity values based on weighted-UniFrac distances. **f** Heat map of selected most differentially abundant features at the family level between WT and TLR4^−/−^ mice. **g** Heat map of selected most differentially abundant features at the genus level between WT and TLR4^−/−^ mice

High-throughput gene sequencing of 16S rRNA in faecal bacterial DNA isolated from WT and TLR4^−/−^ mice was conducted. Different alpha-diversity indices, including Simpson-reciprocal (*p* = 0.0019), Shannon (*p* = 0.0017), Chao (*p* = 0.0001), and observed features (*p* = 0.0019), displayed similar tendencies, indicating that WT mice harboured a microbiota with a distinct diversity compared with TLR4^*−*/*−*^ mice (Fig. 2a).

Principal coordinate analysis (PCoA) using the Bray-Curtis metric distance and weighted UniFrac distance algorithms was performed to evaluate the beta diversity. An apparent clustering separation between amplicon sequence variants (ASVs) revealed the difference in community structures between WT and TLR4^−/−^ mice (Fig. 2b and d). Comparison of within- and between-group dissimilarity analyses revealed that the microbiome difference between WT and TLR4^−/−^ mice was greater than the difference within each genotype (Fig. 2c, calculated from Fig. 2b; Fig. 2e, calculated from Fig. 2d). We assessed the general landscape of the gut microbiota in all available samples to further investigate the potential compositional differences at various taxonomic levels (Fig. S2 a–c). The intestinal flora composition between different genotypes can be segregated by a comparison heatmap based on the ASV abundance at both the family (Fig. 2f) and genus levels (Fig. 2g). Lactobacillaceae (the family and genus *Lactobacillus*) and Akkermansiaceae (the family and genus *Akkermansia*) displayed a significant enrichment in WT mice, while Rikenellaceae (the family and genus *Rikenella*) showed a relatively high abundance in TLR4^−/−^ mice. Collectively, these results demonstrated that the intestinal microbiome in WT mice is obviously differed from TLR4^−/−^ mice.

### A predisposing microbiota in TLR4^−/−^ mice is responsible for the enhanced susceptibility to colitis

To investigate whether the gut microbiota in TLR4^−/−^ mice was responsible for the enhanced susceptibility to colitis, we performed FMT experiments in which pseudosterile WT or TLR4^−/−^ recipient mice were reconstructed with the microbiome from WT or TLR4^−/−^ donor mice (Fig. S3a). FM(WT)→WT or FM(WT)→TLR4^−/−^ resulted in significantly less body weight loss (Fig. S3b), better survival (Fig. S3c), higher DAI scores (Fig. S3d) and histology scores (Fig. S3 g and h), and a greater colon length than FM(TLR4^−/−^)→WT or FM(TLR4^−/−^)→TLR4^−/−^ (Fig. S3 e and f). Additionally, FM(WT)→WT and FM(WT)→TLR4^−/−^ mice displayed comparable weight loss, survival, disease index, colon length, and histology score. FM(TLR4^−/−^)→WT and FM(TLR4^−/−^)→TLR4^−/−^ mice exhibited similar colitis phenotypes (Fig. S3). Consistent with the phenotype, FM(WT)→TLR4^−/−^ and FM(WT)→WT mice harboured similar intestinal flora structures following faecal transfer from WT donors, while FM(TLR4^−/−^)→WT and FM(TLR4^−/−^)→TLR4^−/−^ mice exhibited similar taxonomic community compositions based on beta diversity measurements (Fig. S4 a–c, Fig. S5 a–c).

**Fig. 3:**
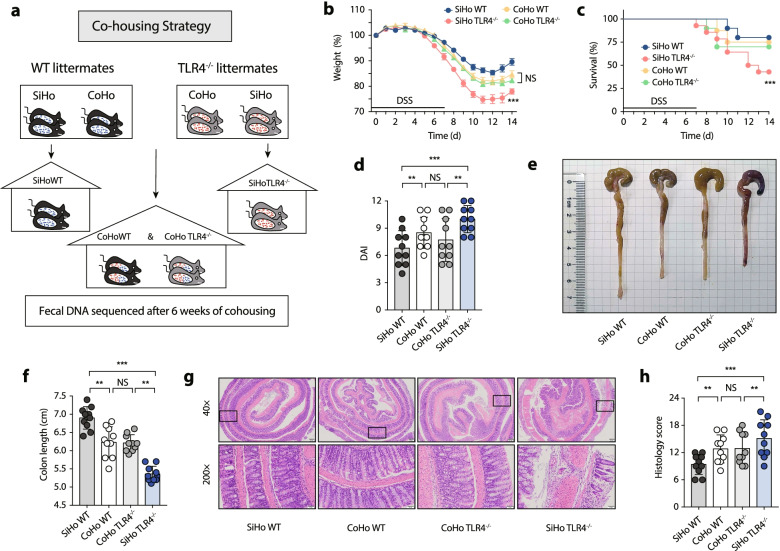
Attenuation of colon inflammation in TLR4^−/−^ mice after co-housing. **a** CoHo-SiHo strategy. **b** Body weight change. **c** Survival. **d** DAI score. **e** Representative pictures of colon gross appearance. **f** Colon length. **g** Representative microscopic pictures of H&E staining (40× and 200× magnification). **h** Histology score. **b**–**h** *n* = 10 mice per group, mean values ± SEM are presented, *p*-values were calculated using two-way analysis of variance (ANOVA) test, *p < 0.05, **p < 0.01, ***p < 0.001. Data are pooled from three independent experiments with *n* = 10 mice per group

Subsequently, spontaneous microbiota transfer studies between different genotypes were performed through cohousing experiments. Age- and sex-matched WT and TLR4^−/−^ littermates were either housed singly (‘SiHo mice’) or cohoused (‘CoHo mice’) for 6 weeks prior to DSS administration (Fig. 3a). CoHo TLR4^−/−^ mice and their WT cage mates (CoHo WT mice) exhibited a similar phenotype following microbiota exchange through coprophagia, as demonstrated by the corresponding body weight loss (Fig. 3b), survival rate (Fig. 3c), DAI score (Fig. 3d), colon length (Fig. 3e and f), and histology score (Fig. 3g and h). Consistently, the microbiome compositional structure of CoHo TLR4^−/−^ mice was comparable to that of CoHo WT mice, as evidenced by the overlapping coordinates and locations based on PCoA of beta diversity (Fig. S4 d–f, Fig. S6 a–c). Taken together, the predisposing microbiota in TLR4^−/−^ mice was responsible for the enhanced susceptibility to colon inflammation.

### Colonic suppressive RORγt^+^ Treg cells mitigate colitis in a gut microbiota-dependent manner

To investigate the imbalance in immune responses between WT and TLR4^−/−^ mice, the immune status of the intestinal microenvironment was evaluated. The frequency of both subpopulations of colon-resident macrophages [(R1 fraction, CD11b^+^ CD11c^low^ F4/80^+^ CD103^−^ macrophages) and (R2 fraction, CD11b^+^ CD11c^+^ F4/80^+^ CD103^−^ macrophages)] displayed no significant difference between the two genotypes (Fig. S7 a–c). In addition, the population of CD11b^low^ CD11c^+^ F4/80^−^ CD103^+^ (R3 fraction) colonic dendritic cells (DCs) showed a comparable tendency between WT and TLR4^−/−^ mice (Fig. S7 a and d).

**Fig. 4:**
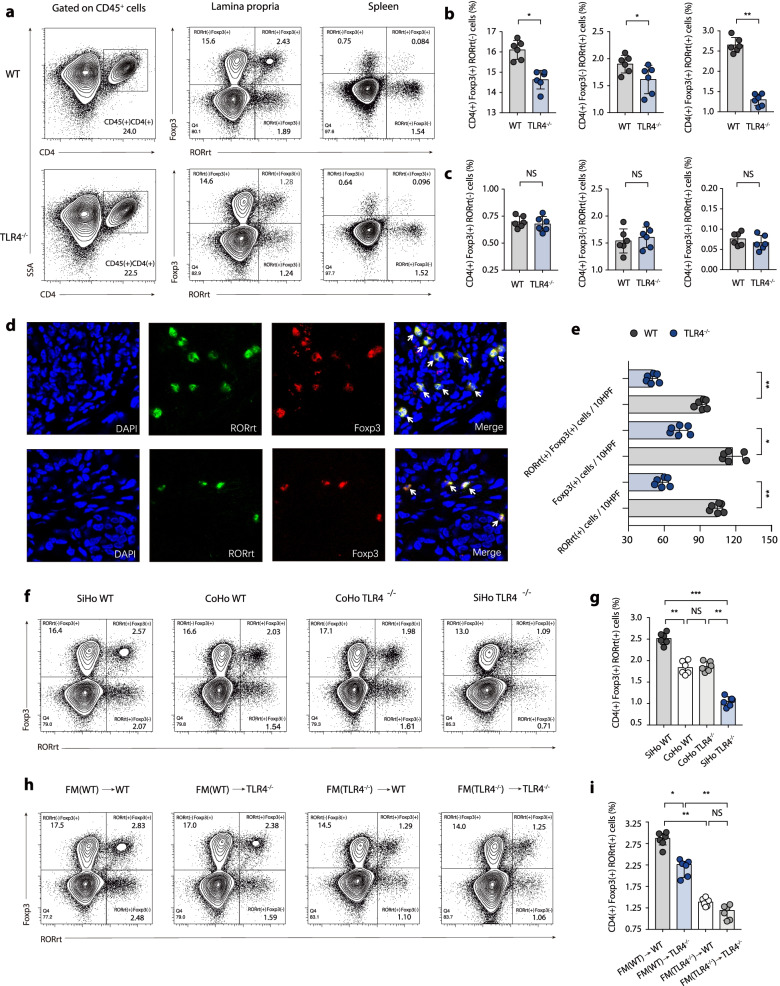
Colonic RORγt^+^ Treg cell-mediated immune responses are gut microbiota-dependent. **a** Representative flow cytometric analysis of colonic LP and spleen RORγt^+^ Treg cells (coexpression of RORγt and Foxp3) in WT and TLR4^−/−^ mice. Numbers in outlined areas indicate percent cells in each gated area. **b** Statistics results of Foxp3^(+)^ RORγt^(−)^ T cells, Foxp3^(−)^ RORγt^(+)^ T cells, Foxp3^(+)^ RORγt^(+)^ T cells in colonic LP in WT and TLR4^−/−^ mice. **c** Statistics results of Foxp3^(+)^ RORγt^(−)^ T cells, Foxp3^(−)^ RORγt^(+)^ T cells, Foxp3^(+)^ RORγt^(+)^ T cells in spleen in WT and TLR4^−/−^ mice. **d** Representative immunofluorescence double staining of RORγt and Foxp3 expression in colon tissue in WT and TLR4^−/−^ mice. **e** Quantification of the total number of double staining cells in ten high-power fields (HPFs) between WT and TLR4^−/−^ mice. **f** Representative flow cytometric analysis of colonic RORγt^+^ Treg cells among SiHo WT, CoHo WT, CoHo TLR4^−/−^, SiHo TLR4^−/−^ groups. **g** Statistical analysis of CD4^(+)^ Foxp3^(+)^ RORγt^(+)^ T cells frequency in co-housing experiments. **h** Representative flow cytometric analysis of colonic RORγt^+^ Treg cells during FMT experiments. **i** Statistical analysis of CD4^(+)^ Foxp3^(+)^ RORγt^(+)^ T cells frequency in FMT experiments. **a**–**e** *n* = 6 mice per group. Data are shown as mean values ± SEM are presented, *p*-values were calculated using unpaired *T*-test, *p < 0.05, **p < 0.01, ***p < 0.001. **f**–**i** *n* = 6 mice per group. Data are shown as mean values ± SEM are presented, *p*-values were calculated using two-way analysis of ANOVA test, *p < 0.05, **p < 0.01, ***p < 0.001. Data are pooled from three independent experiments with *n* = 6 mice per group

Th1 cell responses (Fig. S8 a and b) and Th2 cell responses (Fig. S8 c and d) showed no significant difference between WT and TLR4^−/−^ mice. However, the proportion of Th17 cells (Fig. S8 e and f) and Treg cells (Fig. S8 g and h) in the colonic lamina propria (LP) of TLR4^−/−^ mice was significantly decreased compared with that in WT mice, which was inconsistent with the phenotype results. To better characterize Th17/Treg balance transformation-associated T-cell responses, we analysed the expression of both RORγt and Foxp3 in CD4^+^ T cell subsets. The abundance of RORγt^+^ Treg cells (coexpression of RORγt and Foxp3) in WT mice manifested a pronounced enrichment in comparison with TLR4^−/−^ mice (WT vs. TLR4^−/−^, 2.65% vs. 1.30%; *p* < 0.01) (Fig. 4a and b). The cytokine profile analysis of Th17 and Treg cells has also been analysed (Fig. S9 a and b). Increasing evidence indicates that RORγt^+^ Treg cells represent a stable regulatory T-cell effector lineage, with reinforced anti-inflammatory and immune-suppressing effects during colitis [21, 22]. Given the immunological crosstalk between the gut and other tissue compartments, we investigated whether the observed differences in RORγt^+^ Treg cell responses in the gut could be detected more systemically. However, RORγt^+^ Treg cells were not found in peripheral lymphoid organs (such as the spleen) (Fig. 4a and c). Additionally, we confirmed the RORγt and Foxp3 expression in colon tissue using immunofluorescence double staining, and coexpression of RORγt and Foxp3 in T-cell subsets was significantly higher in WT than in TLR4^−/−^ mice (Fig. 4d and e). Correlation analysis results showed that the ratio of suppressive RORγt^+^ Treg cells in the colonic LP was negatively associated with the colitis phenotype (colon length in Fig. S10a, DAI score in Fig. S10b, histology score in Fig. S10c, colonoscopy score in Fig. S10d).

To illustrate whether the colonic RORγt^+^ Treg cell response was gut microbiota-dependent, the particular T-cell subsets were also phenotyped following faecal transplantation. Consistent with the phenotype in the cohousing experiment, the frequency of RORγt^+^ Treg cells in CoHo TLR4^−/−^ mice was comparable to that of CoHo WT mice (Fig. 4f and g). During the FMT experiment, FM(WT)→WT and FM(WT)→TLR4^−/−^ recipient mice exhibited a similar proportion of colonic RORγt^+^ Treg cells and decreased colon inflammation, while FM(TLR4^−/−^)→WT and FM(TLR4^−/−^)→TLR4^−/−^ recipient mice manifested a comparable abundance of colonic RORγt^+^ Treg cells and aggravated colon inflammation (Fig. 4h and i). These results demonstrated that the frequency of colonic RORγt^+^ Treg cells was negatively correlated with colitis severity, and that suppressive RORγt^+^ Treg cells mitigated colitis in a gut microbiota-dependent manner.

### *Akkermansia muciniphila* is negatively related to the colitis phenotype in a murine model and in UC patient samples

**Fig. 5:**
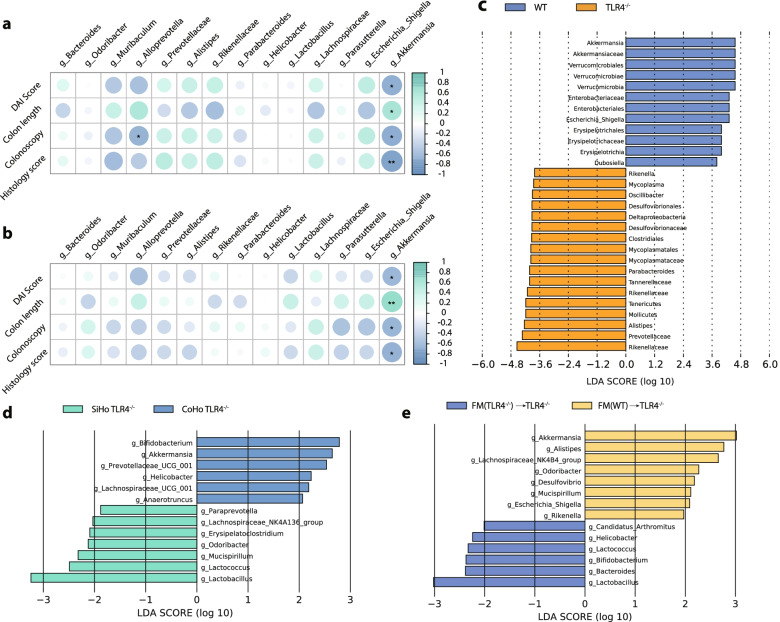
*Akkermansia muciniphila* abundance is negatively related to colitis phenotype. **a** Spearman correlation heat map between differential flora and phenotypic indicators in WT mice. **b** Spearman correlation heat map between differential flora and phenotypic indicators in TLR4^−/−^ mice. **c** LEfSe analysis depicting taxonomic association between microbiome communities from WT and TLR4^−/−^ mice groups. **d** LEfSe analysis depicting taxonomic association between microbiome communities from CoHo TLR4^−/−^ and SiHo TLR4^−/−^ mice groups. **e** LEfSe analysis depicting taxonomic association between microbiome communities from FM(WT)→TLR4^−/−^ mice and FM(TLR4^−/−^)→TLR4^−/−^ mice groups

Correlation analysis between differential flora and phenotypic indicators suggested that the *Akkermansia muciniphila* (*A. muciniphila*) abundance was negatively correlated with the DAI (WT, *R*^2^ = 0.5387; TLR4^−/−^, *R*^2^ = 0.4106), histology (WT, *R*^2^ = 0.6865; TLR4^−/−^, *R*^2^ = 0.5154), and colonoscopy scores (WT, *R*^2^ = 0.5490; TLR4^−/−^, *R*^2^ = 0.4589), and positively correlated with colon length (WT, *R*^2^ = 0.4350; TLR4^−/−^, *R*^2^ = 0.6231) in WT (Fig. 5a, Fig. S11a) and TLR4^−/−^ (Fig. 5b, Fig. S11b) mice. High-dimensional class comparisons using linear discriminant analysis (LDA) of effect size (LEfSe) were conducted to confirm which bacterium was pronouncedly enriched in WT mice and in turn affected disease progression against colon inflammation. Consistently, the *Akkermansia muciniphila* (family Akkermansiaceae; order Verrucomicrobiales; class Verrucomicrobiae; phylum Verrucomicrobia) showed a marked predominance in WT mice and reached the highest LDA score of 4.6 (Fig. 5c), in contrast to TLR4^−/−^ mice.

The microbiome taxonomic comparison heat map (Fig. S12a), combined with the LEfSe analysis (Fig. 5d), showed that *A. muciniphila* was notably predominant in CoHo TLR4^−/−^ mice after cohousing. The *A. muciniphila* abundance was modestly higher in CoHo TLR4^−/−^ mice than in SiHo TLR4^−/−^ mice and was associated with mitigated colon inflammation in CoHo TLR4^−/−^ mice. FMT sequencing results illustrated that FM(WT)→TLR4^−/−^ mice exhibited a slight enrichment of *A. muciniphila* compared with FM(TLR4^−/−^)→TLR4^−/−^ mice (Fig. 5e, Fig. S12b). In general, the microbiome transferred from WT mice, especially the predominant bacterium *A. muciniphila*, ameliorated disease susceptibility in TLR4^−/−^ mice.

To determine the correlation between *A. muciniphila* and chronic colon inflammation, 16S rRNA gene high-throughput sequencing was conducted in faecal bacterial DNA isolated from paired UC patients and healthy controls. PCoA revealed an apparent clustering separation between UC patients and healthy participants in terms of the microbiome structures (Fig. S13a). Remarkably altered bacterial strains were identified by a comparison heat map of UC patients with healthy participants, and *A. muciniphila* abundance was significantly downregulated in patients with UC (Fig. S13b). LEfSe analysis also indicated that *A. muciniphila* was the predominant biomarker in healthy participants compared with UC patients (Fig. S13c). Additionally, we downloaded the published raw 16S rRNA gene sequencing data [23] from the open-source microbiome deposition site QIITA (https://qiita.ucsd.edu/) under study ID 1939. Microbiome sequencing of 16S rRNA obtained from stool samples (Fig. S14 a and b) or mucosal biopsies of different intestinal locations (rectum in Fig. S15 a and b, colon in Fig. S16 a and b, terminal ileum in Fig. S17 a and b) of UC patients revealed a decreased abundance of *A. muciniphila* compared with the healthy population. Taken together, the *A. muciniphila* abundance was negatively related to colitis risk, not only in murine models but also in UC patient samples.

### *Akkermansia muciniphila* supplementation suppresses colon inflammation and increases the frequency of colonic RORγt^+^ Treg cells

**Fig. 6:**
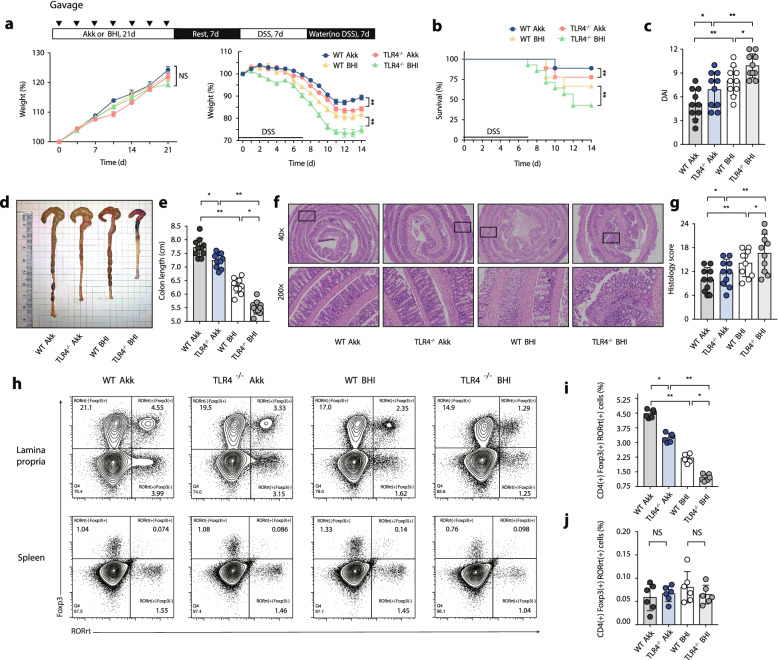
*A. muciniphila* administration suppresses colon inflammation both in WT and TLR4^−/−^ mice through regulating T-cell-associated immune responses. After incubation with *A. muciniphila* or BHI for 21 days, mice were consistently given oral administration of 2.5% DSS for 7 days followed by normal water drinking for a further 7 days. **a** Body weight change. **b** Survival. **c** DAI score. **d** Representative pictures of colon gross appearance. **e** Colon length. **f** Representative microscopic pictures of H&E staining (40× and 200× magnification). **g** Histology score. **h** Representative flow cytometric analysis of colonic and spleen RORγt^+^ Treg cells among WT Akk, TLR4^−/−^ Akk, WT BHI, TLR4^−/−^ BHI groups. Numbers in outlined areas indicate percent cells in each gated area. **i** Statistical analysis of CD4^(+)^ Foxp3^(+)^ RORγt^(+)^ T cells frequency in colonic LP among groups. **j** Statistical analysis of CD4^(+)^ Foxp3^(+)^ RORγt^(+)^ T cells frequency in spleen among groups. **a**–**g** *n* = 10 mice per group, **h**–**j** *n* = 6 mice per group, mean values ± SEM are presented, *p*-values were calculated using two-way analysis of ANOVA test, *p < 0.05, **p < 0.01, ***p < 0.001. Data are pooled from three independent experiments

We inoculated WT and TLR4^−/−^ mice with *A. muciniphila*, and colonization was identified by 16S rRNA sequencing (Fig. S18). Mice that were administered *A. muciniphila* ([WT Akk] or [TLR4^−/−^ Akk]) and control mice that received the brain-heart infusion (BHI) vehicle ([WT BHI] or [TLR4^−/−^ BHI]) had similar body weights before DSS treatment (Fig. 6a). Strikingly, [WT Akk] and [TLR4^−/−^ Akk] mice exhibited decreased colon inflammation, including reduced body weight loss (Fig. 6a), decreased mortality (Fig. 6b), lower DAI scores (Fig. 6c), lower colon histopathology scores (Fig. 6f and g), and greater colon lengths (Fig. 6d and e) than [WT BHI] or [TLR4^−/−^ BHI] mice.

Previous studies have indicated that *A. muciniphila* induces gut microbiota remodelling and activates anti-inflammatory Treg cell responses [23, 24]. In our study, colonic tissue Foxp3 and RORγt expression were both positively correlated with *A. muciniphila* colonization (Fig. S19 a and b), suggesting a potential induction of Foxp3 and RORγt expression by *A. muciniphila*. Consistent with the phenotype, *A. muciniphila* administration significantly increased colonic RORγt^+^ Treg cell responses compared with the BHI vehicle controls (Fig. 6h and i), and *A. muciniphila* supplementation did not change the frequency of RORγt^+^ Treg cells in the mouse spleen (Fig. 6h and j). Colonic innate immune responses were also phenotyped, and *A. muciniphila* administration did not significantly transform macrophage and DC immune responses (Fig. S20 a–d).

**Fig. 7:**
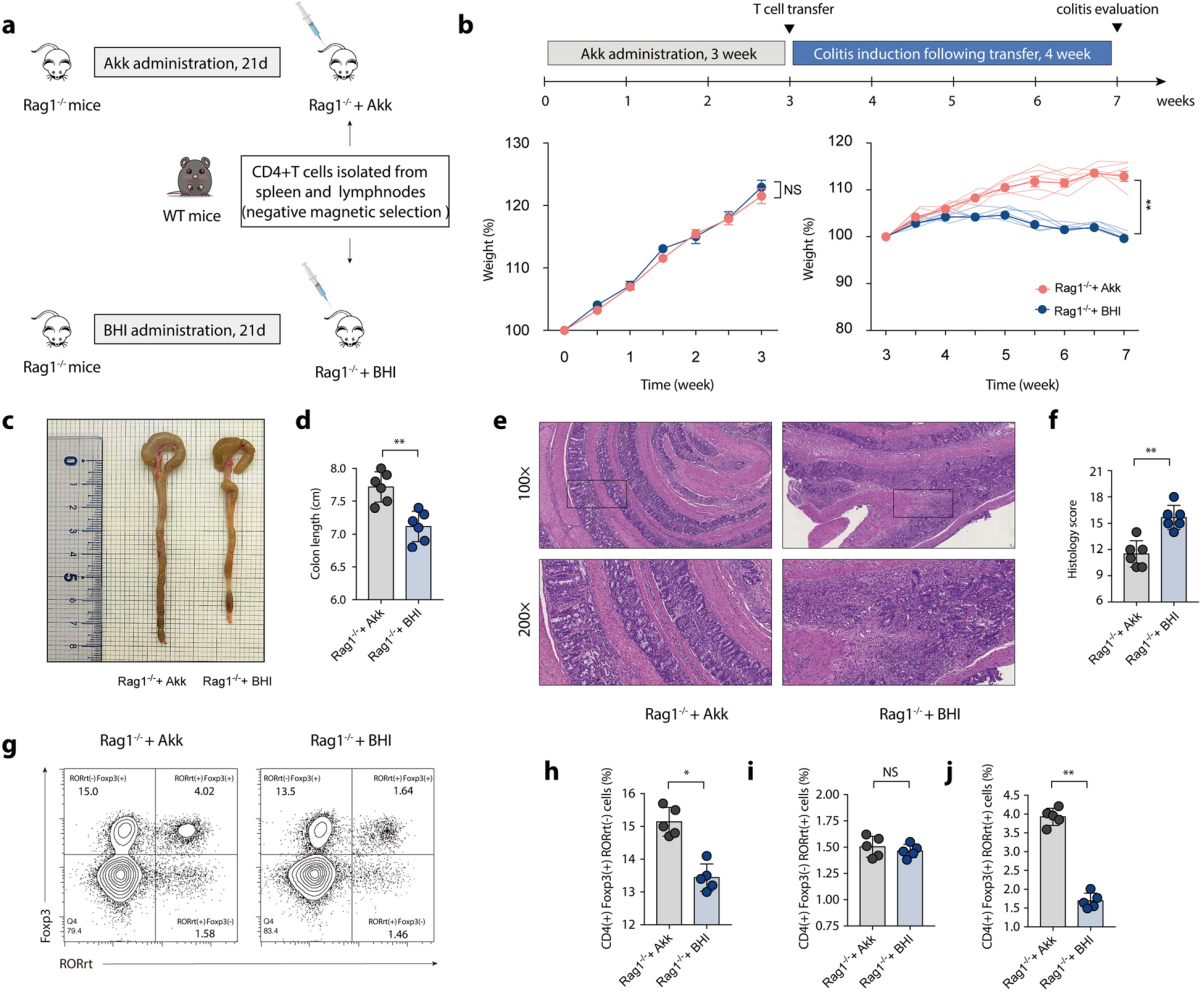
*A. muciniphila* downregulate the colitis predisposition in susceptible mice through induction of suppressive RORγt^+^ Treg cells. Colitis was induced by transferring CD4^+^ T cells into Rag1-deficient (Rag1^−/−^) mice colonized with *A. muciniphila* or BHI medium. **a** T-cell transfer strategy. **b** Body weight change. Thin lines represent the mean data from a group of 6 mice, and bold lines represent the mean ± SEM of all groups of mice colonized either with *A. muciniphila* or BHI medium. **c** Representative pictures of colon gross appearance. **d** Colon length. **e** Representative microscopic pictures of H&E staining (100× and 200× magnification). **f** Histology score. **g** Representative flow cytometric analysis of colonic RORγt^+^ Treg cells between (Rag1^−/−^ + Akk) and (Rag1^−/−^ + BHI) mice. **h** Statistics results of Foxp3^(+)^ RORγt^(−)^ T cells frequency. **i** Statistics results of Foxp3^(−)^ RORγt^(+)^ T cells frequency. **j** Statistics results of Foxp3^(+)^ RORγt^(+)^ T cells frequency. **a**–**f** *n* = 6 mice per group, **g**–**j** *n* = 5 mice per group, mean values ± SEM are presented, *p*-values were calculated using unpaired *T*-test, *p < 0.05, **p < 0.01, ***p < 0.001. Data are pooled from three independent experiments

To demonstrate the specific immune regulation of RORγt^+^ Treg cells by *A. muciniphila*, a T-cell transfer model, which is dependent on both T cells and microbiota, was conducted in our study. At 3 weeks prior to the T-cell transfer treatment, we inoculated Rag1-deficient (Rag1^−/−^) mice with *A. muciniphila* or BHI medium via oral gavage for 3 weeks (Fig. 7a). Mice colonized with *A. muciniphila* or BHI medium had comparable initial body weights before T-cell transfer (Fig. 7b). Colon inflammation evaluation was conducted 4 weeks following T-cell transfer treatment. As measured by body weight loss (Fig. 7b), colon length (Fig. 7c and d), and histology score (Fig. 7e and f), colon inflammation was more severe in mice colonized with BHI (Rag1^−/−^ + BHI) than in mice colonized with *A. muciniphila* (Rag1^−/−^ + Akk). In particular, *A. muciniphila* supplementation notably elevated the ratio of colonic RORrt^+^ Treg cells in Rag1^−/−^ + Akk mice (Fig. 7g–j).

**Fig. 8:**
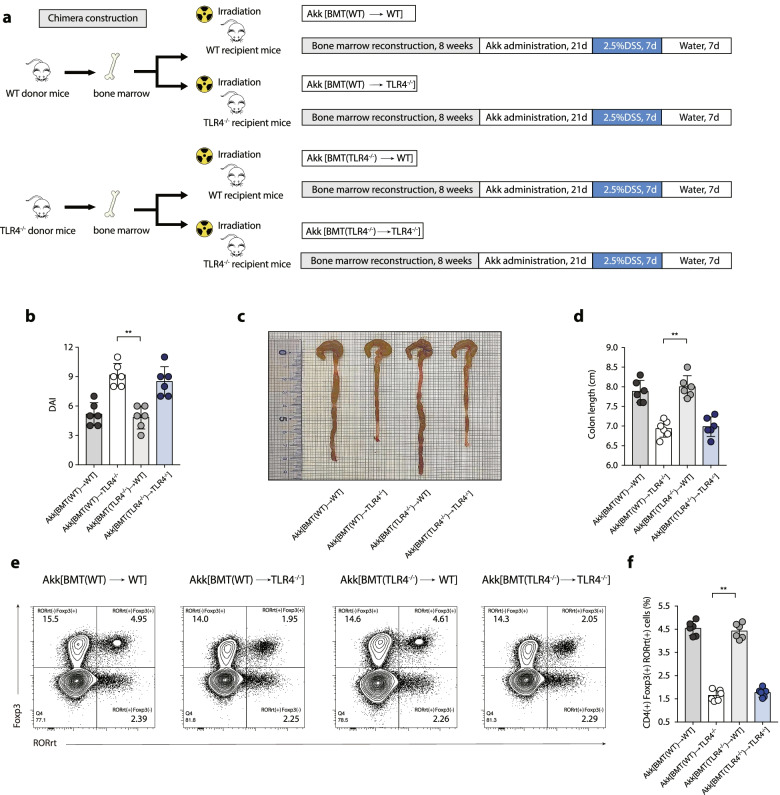
Mitigated colitis and increased frequency of RORγt^+^ Treg cells in BMT chimeras following *A. muciniphila* incubation. **a** BMT chimeras were incubated with *A. muciniphila* for 21 days before 2.5% DSS administration. **b** DAI score. **c** Representative pictures of colon gross appearance. **d** Colon length. **e** Representative flow cytometric analysis of colonic RORγt^+^ Treg cells among BMT chimeras following *A. muciniphila* incubation. **f** Statistical analysis of CD4^(+)^ Foxp3^(+)^ RORγt^(+)^ T cells frequency. **a**–**f** *n* = 6 mice per group, mean values ± SEM are presented, and *p*-values were calculated using two-way analysis of ANOVA test, *p < 0.05, **p < 0.01, ***p < 0.001. Data are pooled from three independent experiments

To investigate immune regulation through intestinal epithelial cell or bone marrow-derived cell TLR4 pathways, bone marrow chimaeras were generated by lethally irradiating WT and TLR4^−/−^ recipient mice to eliminate haematopoietic stem cells, and the haematopoietic compartment was reconstituted by bone marrow transplant (BMT) (Fig. S21a). Flow cytometry phenotyping results indicated that BMT(TLR4^−/−^) →WT manifested an increased frequency of colonic RORrt^+^ Treg cells compared with BMT(WT) →TLR4^−/−^ (2.11 ± 0.05% vs. 1.13 ± 0.03%, *p* < 0.01) (Fig. S21 b and c). The BMT results indicated that the intestinal epithelial-derived TLR4 pathway participated in the intestinal immune activation against colon inflammation. Another set of chimaeras was incubated with *A. muciniphila* intragastrically for 3 weeks after bone marrow reconstitution (Fig. 8a), and *A. muciniphila* colonization was identified by 16S rRNA sequencing (Fig. S22). Colitis was more severe in Akk[BMT(WT)→TLR4^−/−^] than in Akk[BMT(TLR4^−/−^)→WT] after 2.5% DSS administration, as measured by the DAI score (Fig. 8b) and colon length (Fig. 8c and d). Consistent with the phenotype, Akk[BMT(TLR4^−/−^)→WT] displayed a marked enrichment of colonic RORrt^+^ Treg cells compared with Akk[BMT(WT) →TLR4^−/−^](4.42 ± 0.13% vs. 1.65 ± 0.10%, *p* < 0.01) (Fig. 8e and f). Collectively, *A. muciniphila* administration suppressed colitis by activating colonic RORγt^+^ Treg cell-mediated immune responses.

### The interaction between TLR4 and Amuc-1100 mediated colonization of *A. muciniphila*

Considering the decreased abundance of *A. muciniphila* in TLR4^−/−^ mice, we proposed that TLR4 might affect the intestinal colonization of *A. muciniphila*. Colon tissue from both healthy participants and WT mice indicated that TLR4 expression was positively correlated with *A. muciniphila* colonization during gut homeostasis (Fig. S23 a and b). FISH staining of colonic sections from WT mice revealed increased hybridization of the *A. muciniphila* probe MUC1437 [25, 26] compared with those from TLR4^−/−^ mice (Fig. S23c). Based on the genomic and proteomic analysis of *A. muciniphila*, the pili-like external membrane protein *Amuc-1100* might participate in the colonization of *A. muciniphila* [27, 28]*.* Subsequently, we downloaded the molecular model of TLR4 (Fig. S24a) and also constructed the 3D structure model of protein *Amuc-1100* (https://zhanglab.ccmb.med.umich.edu/I-TASSER/) on the basis of its amino acid sequence (Fig. S24b). The configurations of TLR4 and *Amuc-1100* were simulated through Z-DOCK, and the 10 top-ranked possible complex scenarios were displayed (Figs. S25–27). According to the energy-based scoring system, the complex 2 manifested the most likely complex scenario one (Fig. S24 c and d). Collectively, our results indicated that the interaction between TLR4 and *Amuc-1100* might mediate the intestinal colonization of *A. muciniphila*.

## Discussion

Well-balanced interactions between gut microbiota and host immune systems are essential to maintain intestinal homeostasis. As a key member of the PRR family, TLR4 mediates the crosstalk and interplay between the intestinal commensal microbiome and host immune systems. In this study, we investigated the protective role of TLR4 in the shaping of colonic bacterial compositions and microbiota-associated immunity against colon inflammation. Genetic deletion of TLR4 has drastic consequences on the structure and composition of the intestinal microbial communities, leading to a shift towards a proinflammatory configuration that drives enhanced susceptibility and vulnerability to colitis. The decreased abundance of the predominant bacterium *A. muciniphila* and the reduced proportion of suppressive RORγt^+^ Treg cells in TLR4^−/−^ mice contribute to the enhanced susceptibility to colon inflammation. Therefore, our results demonstrate that TLR4 is an indispensable regulator and keeper of gut homeostasis.

The gastrointestinal tract is populated by trillions of diverse and complex microorganisms that function as key players in energy metabolism and host susceptibility to multiple intestinal conditions and diseases [29, 30]. Previous studies have indicated that TLR4 deficiency renders mice susceptible to DSS-induced colitis, which is associated with impaired intestinal barrier function and changes in the inflammatory cytokine profile [18, 20]. Unlike conventionally raised TLR4^−/−^ mice, which are more susceptible to colitis than WT mice, ABX(TLR4^−/−^), mice exhibited indistinguishable colon inflammation following broad-spectrum antibiotic treatment for gut microbiota deprivation in our study when compared with ABX(WT) mice. Our results indicated that resident intestinal bacteria were required for the enhanced susceptibility of TLR4^−/−^ mice to colitis. Gut microbiota dysbiosis, which is usually characterized by the loss of beneficial commensal microflora, expansion of pathogenic bacteria, and reduced overall biodiversity of the microbial ecosystem, is associated with the pathogenesis of IBD [31, 32]. These compositional abnormalities and structural alterations could be both the cause and consequence of IBD, thus inducing a vicious cycle of persistent inflammatory responses. Previous studies have shown that a deficiency in NOD2 or NLRP6 results in a colitogenic microbiota that can exacerbate DSS-induced colitis, and the aggravated colitis phenotype exhibited by NOD2^−/−^ or NLRP6^−/−^ mice could be transferred to WT mice by cohousing [33, 34]. Our cohousing and bacteria-transfer experiments collectively demonstrate that dysbiosis and exacerbated colitis caused by the loss of TLR4 can be partially reversed by transferring gut microbiota from WT mice, further supporting our conclusion that TLR4 is functionally important for the maintenance of intestinal homeostasis and that its deficiency shapes a transmissible, disease-predisposing intestinal microflora.

IBD is characterized by unresolved inflammation in the intestinal tract, which is controlled by a complex interplay of innate and adaptive immune mechanisms [35, 36]. Multiple innate and adaptive immune cells and cytokines in time and space orchestrate the development, recurrence, and exacerbation of the imbalanced inflammatory process in IBD [37]. The correlation of the gut microbiome with host immunity involves a bidirectional relationship between microbes and the host innate and adaptive immune systems [4]. The presence of large numbers of symbionts poses an enormous challenge to the host immune system because it must activate robust immune responses to eliminate invading pathogens while maintaining self-tolerance to avoid autoimmune responses against symbiotic flora [38]. Excessive activation of immune systems triggered by bacterial disturbance is recognized as the underlying mechanism of chronic intestinal inflammatory responses in IBD [32]. Accumulating evidence indicates that RORγt^+^ Treg cells represent a stable regulatory T-cell effector lineage with reinforced anti-inflammatory and immune-suppressing effects during intestinal-specific inflammation [39]. Compared with the gut microbiota from healthy donors, transfer of the IBD donor microbiota into germ-free mice resulted in exacerbated colitis accompanied by a significantly decreased proportion of colonic RORγt^+^ Treg cells, suggesting a potential immune-associated mechanism for microbial contribution to IBD pathogenesis [38, 40]. In this study, aggravated colon inflammation in TLR4^−/−^ mice was accompanied by a significantly reduced proportion of RORγt^+^ Treg cells, while mitigated colitis in WT mice was accompanied by a relatively higher frequency of suppressive RORγt^+^ Treg cells. A large body of evidence indicates that intestinal RORγt^+^ Treg cells are highly context-dependent and have functions in promoting host immunity, particularly constraining immunoinflammatory responses under inflammatory conditions [41, 42]. Consistent with our phenotype, CoHo TLR4^−/−^ and FM(WT)→TLR4^−/−^ recipient mice displayed ameliorated colitis and elevated colonic RORγt^+^ Treg cells, indicating that colonic suppressive RORγt^+^ Treg cells mitigated colitis in a gut microbiota-dependent manner.

Among the next-generation beneficial microbes that have been identified, *A. muciniphila* is strongly positioned in the forefront of candidates. Belonging to the *Verrucomicrobia* phylum, the gram-negative anaerobic commensal *A. muciniphila* can degrade mucin and is an abundant member of the human intestinal microbiota [43–45]. *A. muciniphila* is inversely associated with multiple metabolic disorders and chronic inflammation, and colonization with *A. muciniphila* has been reported to have protective effects against high-fat diet (HFD)-induced obesity, facilitate mucosal wound healing, and elevated antitumour responses during anti-PD-1 immunotherapy [46–48]. Consistently, we observed a marked decrease in the enrichment of *A. muciniphila* in stool samples and intestinal tissue biopsies of patients with UC, and *A. muciniphila* abundance was negatively correlated with colitis risk in our animal models. Evidence indicates that *A. muciniphila* administration ameliorates DSS-induced colitis in mice either via microbe-host interactions, which protect gut barrier function and reduce the levels of inflammatory cytokines, or by improving the microbial community [43]. As expected, *A. muciniphila* supplementation markedly ameliorated colitis not only in the WT genotype but also in the TLR4-deficient genotype in our study. In particular, colonic tissue Foxp3 and RORγt expression were both positively correlated with *A. muciniphila* colonization, and *A. muciniphila* administration significantly increased colonic RORγt^+^ Treg cell responses compared with the BHI controls. Studies have shown that *A. muciniphila* induces intestinal adaptive immune responses during homeostasis, and that *A. muciniphila*-specific contextual signals influence T-cell responses to the microbiota and modulate host immune function [49]. Oral administration of *A. muciniphila* induces gut microbiota remodelling in nonobese diabetic (NOD) mice, which is associated with the promotion of Foxp3^+^ regulatory T cells in islets and interleukin 10 and transforming growth factor β in pancreatic lymph nodes [24]. HFD-fed mice administered *A. muciniphila* show improved glucose tolerance and an increased number of adipose tissue-resident CD4^+^ Foxp3 regulatory T cells [50]. Therefore, we hypothesized that *A. muciniphila* could specifically immunoregulate colonic RORγt^+^ Treg cells in our study. A recent study highlighted that mice colonized with IBD microbiota experienced more severe disease and a decreased ratio of RORγt^+^ Treg cells than those colonized with healthy donor microbiota based on a T-cell transfer model of colitis [38]. In our study, susceptible Rag1^−/−^ mice were inoculated with *A. muciniphila* or BHI medium prior to T-cell transfer treatment, and Rag1^−/−^ + Akk mice experienced a mitigated colitis phenotype and an increased percentage of colonic RORrt^+^ Treg cells compared with Rag1^−/−^ + BHI mice, indicating the potential immune induction of colonic RORrt^+^ Treg cells by *A. muciniphila* in a specific context.

Although the beneficial value of *A. muciniphila* as a potential probiotic has been widely recognized, adverse effect may also exist. A prior study demonstrated that the presence of *A. muciniphila* exacerbated the severity of colon inflammation caused by *Salmonella typhimurium* (*S. typhimurium*) infection in mice colonized with a simplified human gut microbiota (SIHUMI) [51]. The presence of *A. muciniphila* alone in mice is not pathogenic; when both *A. muciniphila* and *S. typhimurium* were present in SIHUMI mice simultaneously, colonic inflammation was exacerbated because of the decreased level of IL-18 and macrophage dysfunction. Seregin et al. reported that *A. muciniphila* was sufficient to induce colon inflammation in an IL-10 knockout (IL-10^−/−^) model of colitis [52]. This conflicting conclusion may be explained by various factors, such as the different mouse models used. This phenotype is highly dependent on the genetic background of the specific IL-10 knockout model. Evidence indicated that different commensal bacteria, such as *Escherichia coli* and *Enterococcus faecalis*, could induce immune-mediated inflammation in IL-10^−/−^ mice, but neither bacterial strain caused colon inflammation in wild-type mice [53]. Therefore, the colitogenicity of *A. muciniphila* is context dependent. When gut homeostasis is disrupted, beneficial microbes may switch to potentially virulent species, which may exert harmful effects on the host.

Gene-microbiota interactions contribute to the pathogenesis of IBD, and mutations in genetic pathways linked to IBD result in an inability to sense and/or respond to beneficial microbes [54, 55]. Previous studies demonstrated that NLRP12 deficiency in mice promotes a dysbiotic microbiome, and that NLRP12 attenuates colon inflammation by maintaining colonic microbial diversity and boosting the growth of the protective gut commensal strain (of the family Lachnospiraceae) [56]. Host gene caspase recruitment domain family member 9 (CARD9) affects the composition and function of the gut microbiota, altering the production of microbial metabolites and intestinal inflammation [57]. Evidence indicates that arginase 1 (Arg1) expression impedes the resolution of intestinal inflammation by altering the faecal microbiome and the metabolome [58]. Moreover, unlike pathogens with TLR ligands triggering inflammation, some commensal bacteria exploit the TLR pathway to actively suppress immune reactions [59]. As observed in the interplay between *A. muciniphila* and suppressive RORγt^+^ Treg cells in our study, TLR4 is required for shaping the colonic ecology and maintaining intestinal homeostasis. Correlation analysis results indicate that TLR4 expression is positively related to *A. muciniphila* colonization in both human and mouse samples. Combined with our FISH staining results showing that intestinal *A. muciniphila* colonization manifested significant accumulation during homeostasis in WT mouse samples, we hypothesized that TLR4 could exert an influence on the intestinal colonization of *A. muciniphila*. Genomic and proteomic analysis of *A. muciniphila* indicated that external membrane protein *Amuc-1100* might participate in the colonization process [27, 28], and our docking results also demonstrated the interaction between TLR4 and *Amuc-1100*. Further in-depth mechanisms will be explored in future research.

## Conclusions

In conclusion, our study identified an unexpected role for TLR4 in regulating intestinal microbiota compositions and susceptibility to colon inflammation through shaping the colonization of *A. muciniphila*. In particular, *A. muciniphila*-based mechanisms play a fundamental role in driving the divergent induction of suppressive RORγt^+^ Treg cells in the gut-specific microenvironment. Therefore, based on the interplay and crosstalk between gut microbiota and immune responses, our results may offer alternative avenues for therapeutic intervention in IBD.

## Methods

### Patients

All participants were recruited via the Department of Gastroenterology, The Second Affiliated Hospital of Third Military Medical University. Participants were excluded if they had used antibiotics, sulfasalazine, probiotics, or prebiotics in the month preceding faecal sampling, as this could influence the intestinal microbiota composition and structure. We collected faecal samples from 29 UC patients and 35 age- and sex-matched healthy controls. Both patients and healthy subjects were Chongqing habitants and consumed an eastern diet. Intestinal biopsy specimens were collected from 62 healthy participants who underwent colonoscopy for dyspeptic symptoms at the Digestive Endoscopy Center of The Second Affiliated Hospital of Third Military Medical University. The research group strictly followed the guidelines of the Declaration of Helsinki and the principle of biomedical research involving human norms of international ethics established by the WHO and CIOMS. The study was approved by the Medical Ethics Committee of The Second Affiliated Hospital of Third Military Medical University (No. AMWUEC2020582). Written informed consent was provided by all participants before collection of the samples.

### Mice

All animal experimental protocols were performed following the guidelines of the National Institutes of Health Guide for the Care and Use of Laboratory Animals and approved by the Laboratory Animals Welfare and Ethics Committee of Third Military Medical University (No. AMWUEC2020582). TLR4-knockout (TLR4^−/−^) mice on the C57BL/6J background were obtained from GemPharmatech (Co., Ltd., Jiangsu, China). Both TLR4^−/−^ mice and wild-type (WT) C57BL/6J mice were originally generated from the same breeders and house raised at the Animal Center of Third Military Medical University Second Affiliated Hospital for at least ten generations. Age- and sex-matched WT and TLR4^−/−^ littermates were employed in our study. All animals were maintained in the same room and generally in the same facilities (such as racks and litters). They were maintained by the same personnel, who changed their gloves and used sterile utensils to prevent transfer of microbes between cages. Throughout the acclimatization and study periods, all animals were maintained on a 12-h light-dark cycle (21 ± 2 °C with a relatively constant humidity of 45 ± 10%) under specific pathogen-free (SPF) conditions and had access to food and water ad libitum. All mice were fed irradiated food and maintained in autoclaved cages.

### DSS-induced colitis model

To induce acute experimental colitis, mice were administered 1.5–2.5% (w/v) dextran sodium sulphate (DSS, molecular weight, 36–50 kDa; MP Biomedicals, UK) in their drinking water ad libitum for 7 days. To assess experimental colitis and repair, a recovery model was implemented; specifically, mice were administered 2.5% (w/v) DSS in their drinking water ad libitum for 7 days followed by 7 days of normal water. In all colitis models, mice were checked daily for morbidity, and body weight was recorded. Each mouse was scored for pathological features, including stool consistency, the presence of blood in the stool, and body weight loss. Individual scores were combined to generate the disease activity index (DAI), which was calculated daily for each mouse. The maximum score was 12 based on assigning a 0–4 scoring system for the following parameters: weight loss (0 points = 0% weight loss from baseline; 1 point = 1–5% weight loss; 2 points = 5–10% weight loss; 3 points = 10–20% weight loss; and 4 points = more than 20% weight loss); rectal bleeding (0 points = negative; 2 points = positive hemoccult test; and 4 points = visible bleeding); and stool consistency (0 points = normal faeces; 1 point = loose stool; 2 points = watery diarrhoea; 3 points = slimy diarrhoea, little blood; and 4 points = severe watery diarrhoea with blood).

### T-cell transfer colitis model

T-cell transfer colitis experiments were performed as previously described [38]. Briefly, CD4^+^ T cells were isolated from the spleen and subcutaneous lymph nodes of 6- to 8-week-old SPF C57BL/6J mice. CD4^+^ T cells were enriched using negative magnetic selection (Miltenyi Biotec) following tissue dissociation and red blood cell lysis. Isolated cells were stained with CD4, CD25, and CD45RB, ensuring that CD45RB^high^CD4^+^ T cells accounted for ~50% of the fraction. Immunodeficient Rag1-deficient (Rag1^−/−^) mice (8–10 weeks old, male) on a C57BL/6 background were employed as recipients in our transfer model. Each recipient received an intravenous injection of a minimum of 4 × 10^5^ purified cells (200 μL/injections). Colitis evaluation was conducted 4 weeks following T-cell transfer.

### Histopathology

The colons were emptied of faecal contents, opened longitudinally along the mesenteric border, formed a Swiss roll from the proximal to the distal end, and then placed in 10% neutral buffered formalin for 24 h. The Swiss rolls were transferred to 70% ethanol and then processed into paraffin-embedded blocks to generate 5-μm-thick sections for haematoxylin and eosin (H&E) staining. The sections were evaluated by an experienced pathologist in a blinded manner, and histological scores were assessed according to the following parameters: inflammation, epithelial defects, crypt atrophy, dysplasia/neoplasia, and the area affected by dysplasia. Each parameter generated a separate score with a value between 0 and 4 based on the colon inflammation severity and extent [60, 61].

### Colonoscopy evaluation

Colonoscopy was performed on experimental mice using a high-resolution mouse video endoscopic system (KARL STORZ, Tuttlingen, Germany). The severity of colitis was scored in a blinded manner using MEICS (murine endoscopic index of colitis severity) based on the following 5 parameters: (a) transparency of the colon, (b) changes in the vascular pattern, (c) fibrin visible, (d) granularity of the mucosal surface, and (e) stool consistency [62, 63]. Each parameter was scored with a value between 0 and 3. The cumulative score ranged from 0 (no signs of inflammation) to 15 (signs of very severe inflammation).

### Bone marrow transplantation (BMT)

Recipient mice (8–10 weeks old, male, WT or TLR4^−/−^ mice) received lethal irradiation with a total dose of 11 Gy in two 5.5-Gy fractions separated by 4–5-h intervals. To minimize heterogeneity, bone marrow cells were normally obtained from the long bones of donor mice (8–10 weeks old, male, WT or TLR4^−/−^ mice) with recipient mice of similar age. Harvesting of bone marrow cells for transplant must be done rapidly after death without intervention via chemical means that could complicate the functional status of stem cells. Each recipient mouse typically received 2 × 10^7^ bone marrow cells for engraftment. Peripheral blood was assessed by flow cytometry for the percentage of CD45.1 (donor) and CD45.2 (recipient) leukocytes using standard techniques 4 weeks following transplantation [64]. We generated chimaeras by transferring bone marrow from WT donors or TLR4^−/−^ donors to generate WT mice with myeloid cells deficient in TLR4 (BMT(TLR4^−/−^)→WT) and TLR4^−/−^ mice expressing TLR4 only in myeloid cells (BMT(WT)→TLR4^−/−^), as well as control mice (BMT (TLR4^−/−^)→TLR4^−/−^ or BMT (WT)→WT).

### Immunofluorescence staining

For immunofluorescence staining, colonic segments were embedded in Cryomatrix and frozen on dry ice. Cryosections (10 μm) were fixed for 5 min in 4% paraformaldehyde in PBS, washed in PBS-0.05% Tween 20 (PBT), incubated for 30 min at room temperature in PBT-5% normal goat serum (saturation buffer), and then incubated overnight at 4 °C with primary antibodies diluted in saturation buffer. Sections were counterstained with 4,6-diamidino-2-phenylindole (DAPI) for nuclear staining. Slides were dried and mounted using ProLong Antifade mounting medium (Invitrogen, Molecular Probes, Eugene Oregon). Slides were visualized using a Leica TCS SP5 confocal microscope. The following antibodies were used: AF647-anti-Foxp3 and PE-anti-RORγt. The number of positive cells per field of view under ×800 magnification was counted, and data were collected from ten randomly selected fields.

### Cultivation of *A. muciniphila* and mouse colonization with *A. muciniphila*

*A. muciniphila* MucT (ATCC BAA835) was cultured under strictly anaerobic conditions at 37 °C in brain-heart infusion (BHI) medium as described previously [24, 65], and exponentially growing cultures were washed with PBS and immediately frozen in PBS containing 25% glycerol to a final concentration of 1 × 10^10^ cells per mL [66]. Prior to administration, a frozen pellet of *A. muciniphila* was thawed and resuspended in anaerobic PBS to a concentration of 1.5 × 10^9^ per mL. Mice were treated by oral gavage with a bacterial *A. muciniphila* suspension in BHI twice a week for 3 weeks. BHI broth was used as a vehicle control. After *A. muciniphila* or BHI supplementation, the mice were administered DSS 1 week after the final gavage.

### Bacterial fluorescence in situ hybridization (FISH)

FISH rRNA in situ hybridization was performed on frozen slices according to the FISH kit instructions (BersinBio, Cat. No. QD355). Cryosections were overlaid with 100 μL hybridization buffer [0.9 M NaCl, 0.02 M Tris-HCl (pH 8.0), 0.01% sodium dodecyl sulphate] containing an oligonucleotide mixture (5 ng/μL) consisting of the *A. muciniphila* Cy3-labelled MUC-1437 (5′-CCTTGCGGTTGGCTTCAGAT-3′) and total bacterial FITC-labelled EUB-338 (5′-GCTGCCTCCCGTAGGAGT-3′) probes [25, 26]. Hybridization was conducted at 50 °C for 16 h in a humidified chamber. After hybridization, the tissue sections were washed with a washing buffer (0.02 M Tris-HCl, pH 8, 0.9 M NaCl) for 10 min at 50 °C. Counterstaining was carried out with DAPI, and the slides were visualized with a Leica TCS SP5 confocal microscope equipped with appropriate filter sets.

### Statistical analysis

Statistical analysis was performed with GraphPad Prism 7.0 software (GraphPad Software Inc., San Diego, USA). Significance between two groups was determined using the unpaired, two-tailed Student’s *t*-test, and significance between multiple groups was determined by two-way analysis of variance (ANOVA) with Fisher’s LSD test. The results are shown as the mean ± SEM; statistical significance is indicated as follows: *p < 0.05, **p < 0.01, ***p < 0.001, and NS means no significance.

## Supplementary Information

**Additional file 1: Figure S1**. TLR4^-/-^ mice develop severe DSS-induced colitis. **Figure S2**. Loss of TLR4 significantly alters gut microbiota taxonomic composition. **Figure S3**. FMT alleviates colon inflammation in TLR4^-/-^ mice. **Figure S4**. Gut microbiota PCoA profile of Co-housing and FMT experiments. **Figure S5**. Gut microbiota taxonomic composition in FMT experiments. **Figure S6**. Gut microbiota taxonomic composition in Co-housing experiments. **Figure S7**. The intestinal innate immune responses evaluation between WT and TLR4^-/-^ mice. **Figure S8**. The intestinal adaptive immune responses evaluation between WT and TLR4^-/-^ mice. **Figure S9**. The cytokines profile of Treg and Th17 cells between WT and TLR4^-/-^ mice. **Figure S10**. Correlation analysis between RORγt^+^ Treg cells and clinical parameters. **Figure S11**. Correlation analysis between differential flora and phenotypic indicators. **Figure S12**. Gut microbiota landscope of Co-housing and FMT experiments. **Figure S13**. The relative abundance of *A. muciniphila* is decreased in stool samples in patients with UC. **Figure S14**. The microbiome of UC patients are different from healthy participants. **Figure S15**. The microbiome of UC patients are different from healthy participants. **Figure S16**. The microbiome of UC patients are different from healthy participants. **Figure S17**. The microbiome of UC patients are different from healthy participants. **Figure S18**. *A. muciniphila* abundance discrepancy following single bacteria supplementation. **Figure S19**. Correlation analysis between transcription factor expression and *A. muciniphila* colonization. **Figure S20**. The intestinal innate immune responses evaluation between WT and TLR4^-/-^ mice after *A. muciniphila* supplementation. **Figure S21**. Intestinal epithelial-derived TLR4 pathway participating in intestinal immune activation against colitis. **Figure S22**. *A. muciniphila* abundance discrepancy in BMT experiment following bacteria supplementation. **Figure S23**. TLR4 affects the intestinal colonization of *A. muciniphila* during homeostasis. **Figure S24**. The Interaction between TLR4 and *Amuc-1100* mediated the intestinal colonization of *A. muciniphila*. **Figure S25-S27**. Top-ranked possible complex scenarios of TLR4 and *Amuc-1100* based on ZDOCK prediction. **Supplemental Methods**. Faecal genomic DNA extraction and 16S-rRNA sequencing. Antibiotic cocktail treatment. Co-housing experiment. Faecal microbiota transplantation (FMT). Single-cell isolation. Flow cytometry. Gut microbiota qPCR quantification. Cultivation of *A. muciniphila* and Mouse Colonization with *A. muciniphila*. Meta-analysis of microbiome changes in patients with UC.

## Data Availability

Raw 16S rRNA sequencing data have been deposited in the European Nucleotide Archive (http://www.ebi.ac.uk/ena) with study no. PRJEB44178. The other data are available from the corresponding author upon reasonable request.

## Abbreviations

IBD: Inflammatory bowel disease
CD: Crohn’s disease
UC: Ulcerative colitis
PAMPs: Pathogen-associated molecular patterns
DAMPs: Damage-associated molecular patterns
PRR: Pattern-recognition receptor
TLR4: Toll-like receptor 4
H&E: Haematoxylin and eosin
DSS: Dextran sodium sulphate
DAI: Disease activity index
FMT: Faecal microbiota transplantation
FM: Faecal microbiota
*A. muciniphila*: *Akkermansia muciniphila*
PCoA: Principal coordinate analysis
QIIME: Quantitative insights into microbial ecology
WT: Wild type
ABX: Antibiotic cocktail
GF: Germ-free
BHI: Brain-heart infusion
FISH: Bacterial fluorescence in situ hybridization
