# Supplementary material for: TLR4 regulates RORγt+ regulatory T-cell responses and susceptibility to colon inflammation through interaction with Akkermansia muciniphila
Source: Microbiome. 2022 Jun 27;10:98. doi: 10.1186/s40168-022-01296-x (PMC9235089; doi:10.1186/s40168-022-01296-x)
Supplement: Supplementary file 2 — Additional file 1: Figure S1. TLR4-/- mice develop severe DSS-induced colitis. Figure S2. Loss of TLR4 significantly alters gut microbiota taxonomic composition. Figure S3. FMT alleviates colon inflammation in TLR4-/- mice. Figure S4. Gut microbiota PCoA profile of Co-housing and FMT experiments. Figure S5. Gut microbiota taxonomic composition in FMT experiments. Figure S6. Gut microbiota taxonomic composition in Co-housing experiments. Figure S7. The intestinal innate immune responses evaluation between WT and TLR4-/- mice. Figure S8. The intestinal adaptive immune responses evaluation between WT and TLR4-/- mice. Figure S9. The cytokines profile of Treg and Th17 cells between WT and TLR4-/- mice. Figure S10. Correlation analysis between RORγt+ Treg cells and clinical parameters. Figure S11. Correlation analysis between differential flora and phenotypic indicators. Figure S12. Gut microbiota landscope of Co-housing and FMT experiments. Figure S13. The relative abundance of A. muciniphila is decreased in stool samples in patients with UC. Figure S14. The microbiome of UC patients are different from healthy participants. Figure S15. The microbiome of UC patients are different from healthy participants. Figure S16. The microbiome of UC patients are different from healthy participants. Figure S17. The microbiome of UC patients are different from healthy participants. Figure S18. A. muciniphila abundance discrepancy following single bacteria supplementation. Figure S19. Correlation analysis between transcription factor expression and A. muciniphila colonization. Figure S20. The intestinal innate immune responses evaluation between WT and TLR4-/- mice after A. muciniphila supplementation. Figure S21. Intestinal epithelial-derived TLR4 pathway participating in intestinal immune activation against colitis. Figure S22. A. muciniphila abundance discrepancy in BMT experiment following bacteria supplementation. Figure S23. TLR4 affects the intestinal colonization of A. mucinip [file 40168_2022_1296_MOESM1_ESM.docx]

**supplemental Figures**

**
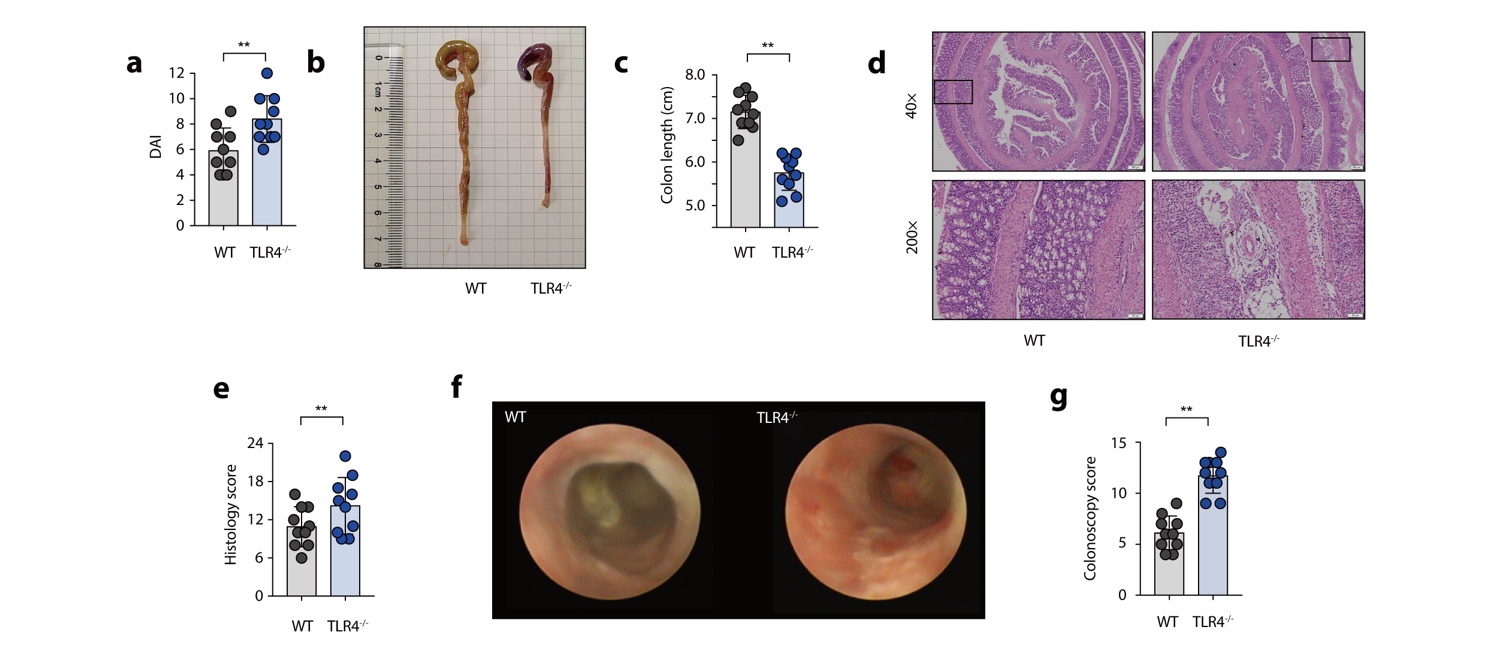
**

**Figure S1. TLR4^-/-^ mice develop severe DSS-induced colitis.**

To assess experimental colitis and repair, age-and-sex matched WT and TLR4^-/-^ mice were given oral administration of 2.5% DSS for 7 days followed by normal water drinking for a further 7 days. (a) Disease activity index (DAI) score. (b) Representative pictures of colon gross appearance. (c) Colon length. (d) Representative microscopic pictures of H&E staining (40× and 200× magnification). (e) Histology score. (f) Representative colonoscopy images. (g) Colonoscopy score.

**
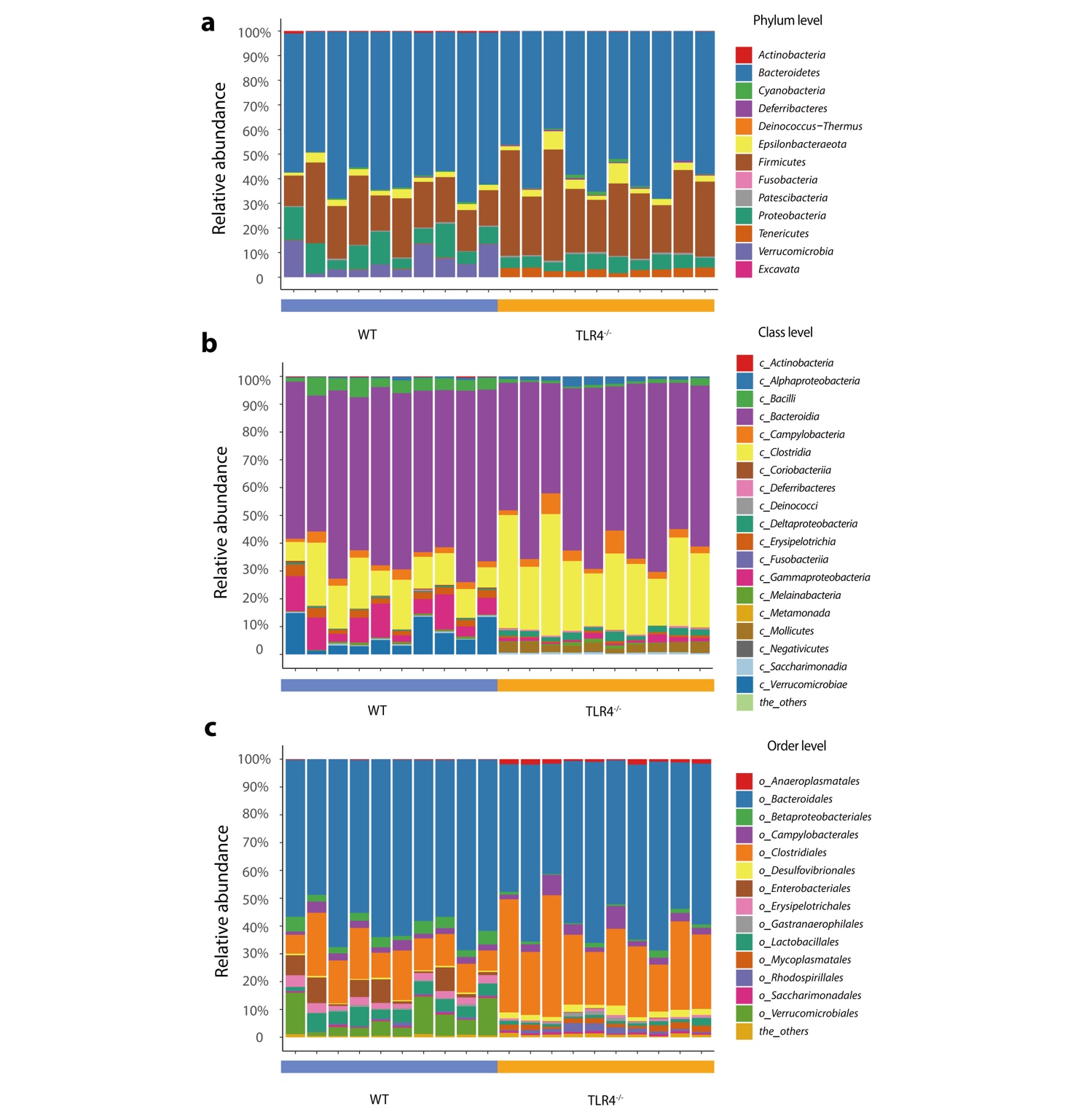
**

**Figure S2. Loss of TLR4 significantly alters gut microbiota taxonomic composition.**

(a) Bar plots of the phylum taxonomic levels in WT and TLR4^-/-^ mice. Relative abundance is plotted for each sample. (b) Bar plots of the class taxonomic levels in WT and TLR4^-/-^ mice. Relative abundance is plotted for each sample. (c) Bar plots of the order taxonomic levels in WT and TLR4^-/-^ mice. Relative abundance is plotted for each sample.

**
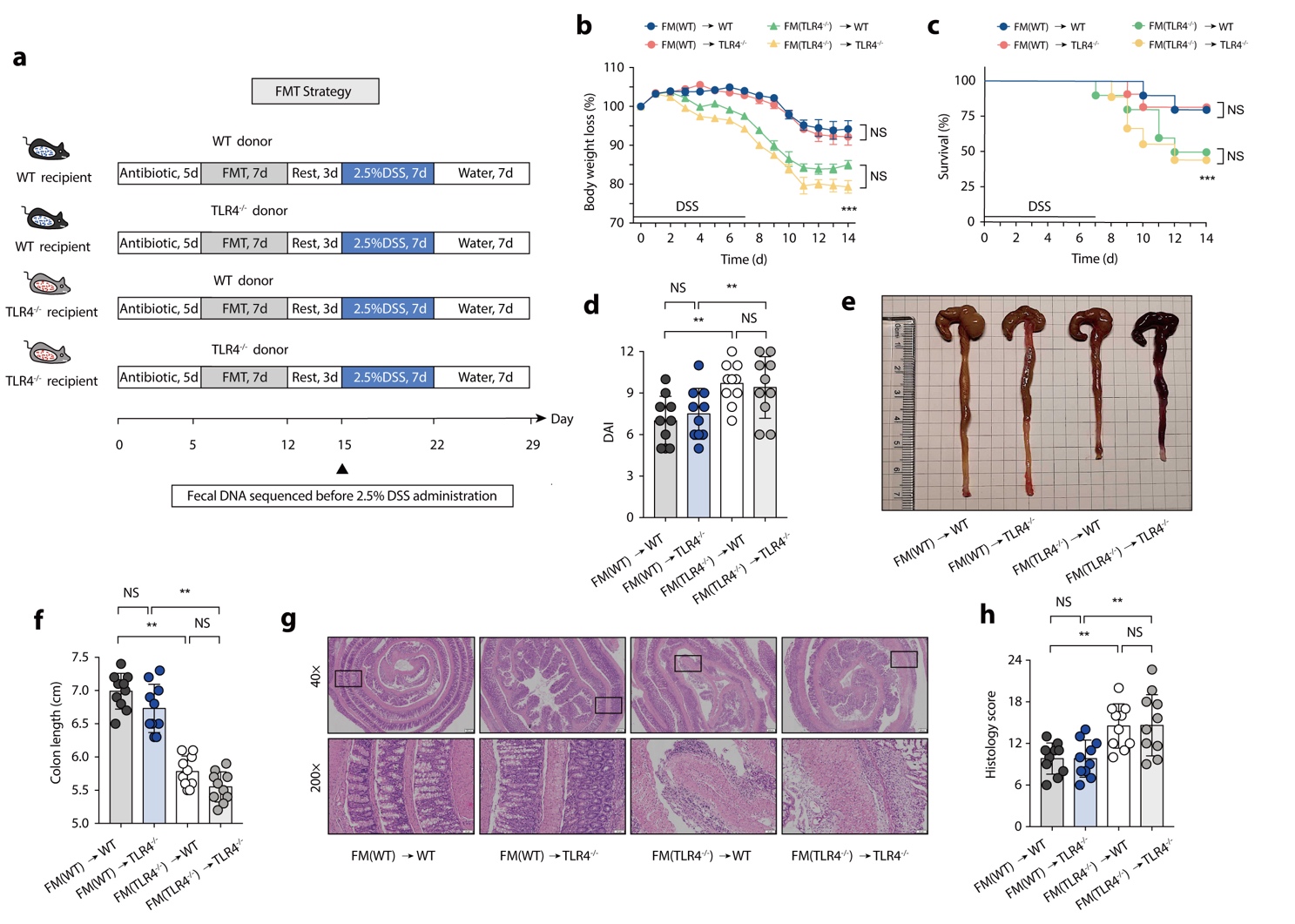
**

**Figure S3. FMT alleviates colon inflammation in TLR4^-/-^ mice.**

(a) FMT strategy. (b) Body weight change. (c) Survival. (d) DAI score. (e) Representative pictures of colon gross appearance. (f) Colon length. (g) Representative microscopic pictures of H&E staining (40× and 200× magnification). (h) Histology score. (a-h) n = 10 mice per group, mean values ± SEM are presented, p values were calculated using two-way analysis of ANOVA test, * p < 0.05, ** p < 0.01, *** p < 0.001. Data are pooled from three independent experiments with n = 10 mice per group.

**
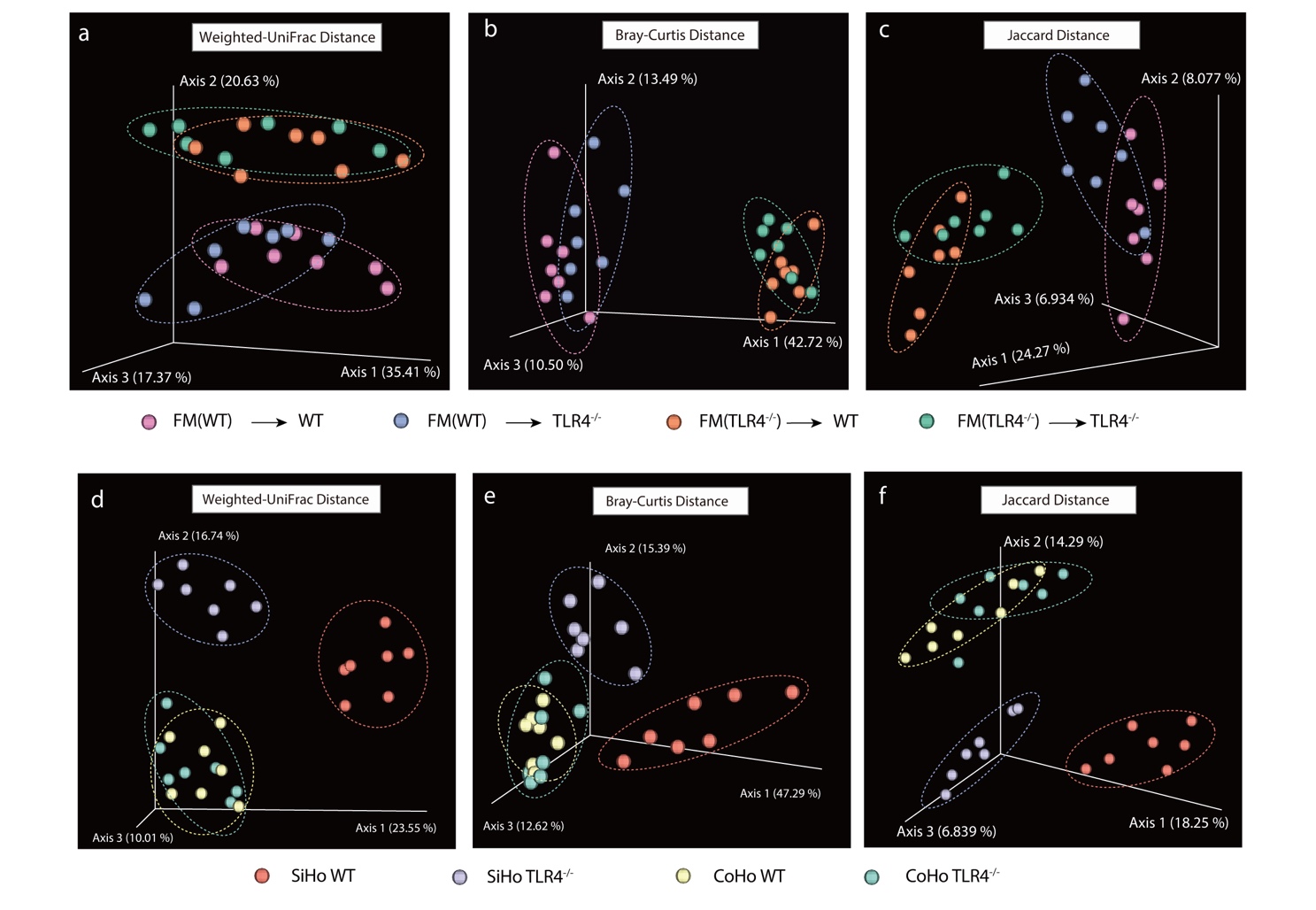
**

**Figure S4. Gut microbiota PCoA profile of Co-housing and FMT experiments.**

(a-c) PCoA of Beta diversity among FMT groups (Based on Weighted-UniFrac distances, Bray-Curtis metric distances and Jaccard distances index). (d-f) PCoA of Beta diversity among SiHo mice (SiHo WT and SiHo TLR4^-/-^) or CoHo mice (CoHo WT and CoHo TLR4^-/-^) groups (Based on Weighted-UniFrac distances, Bray-Curtis metric distances and Jaccard distances index).

**
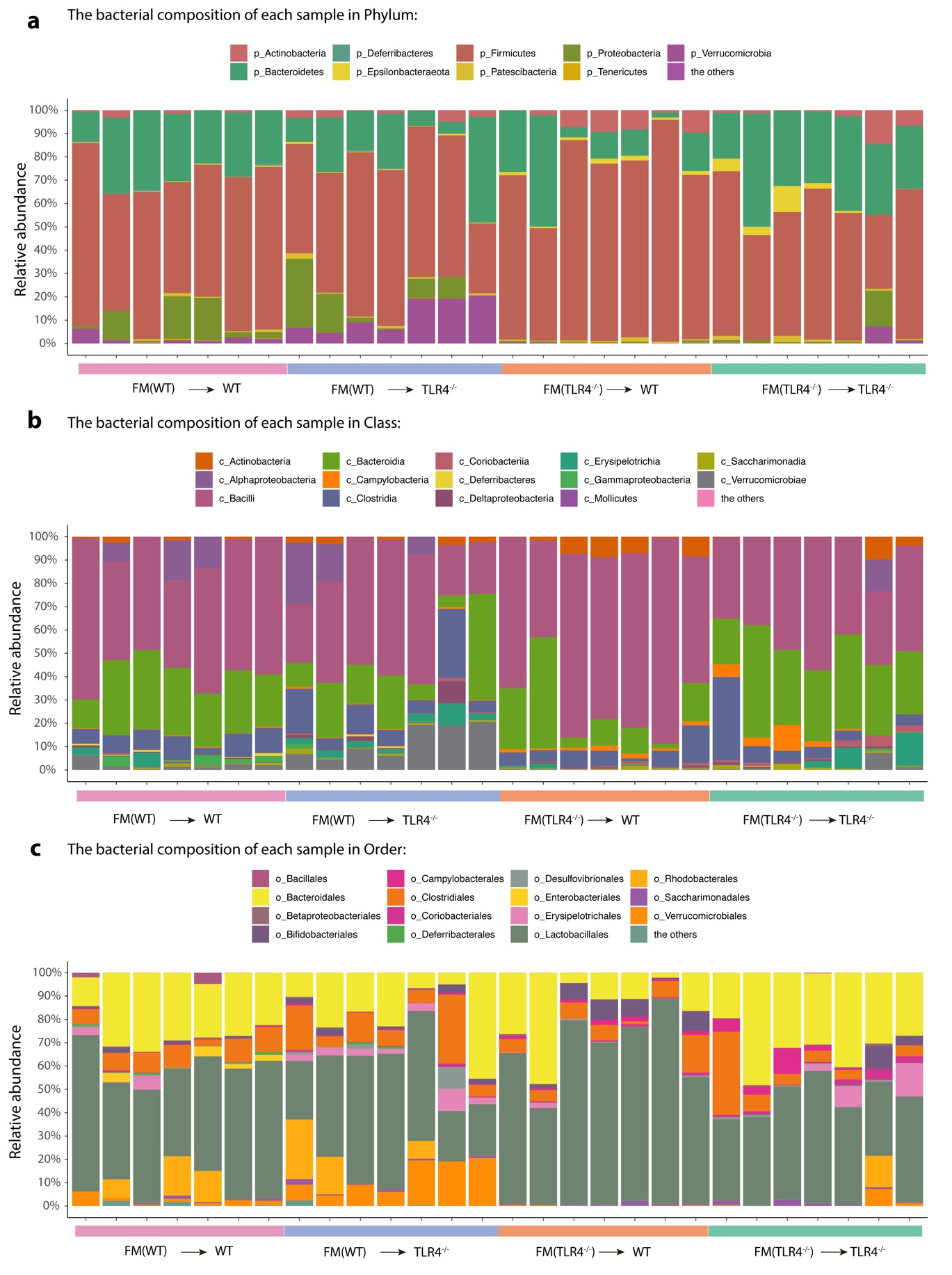
Figure S5. Gut microbiota taxonomic composition in FMT experiments.**

(a) Bar plots of the phylum taxonomic level. (b) Bar plots of the class taxonomic level. (c) Bar plots of the order taxonomic level. Relative abundance is plotted for each sample.

**
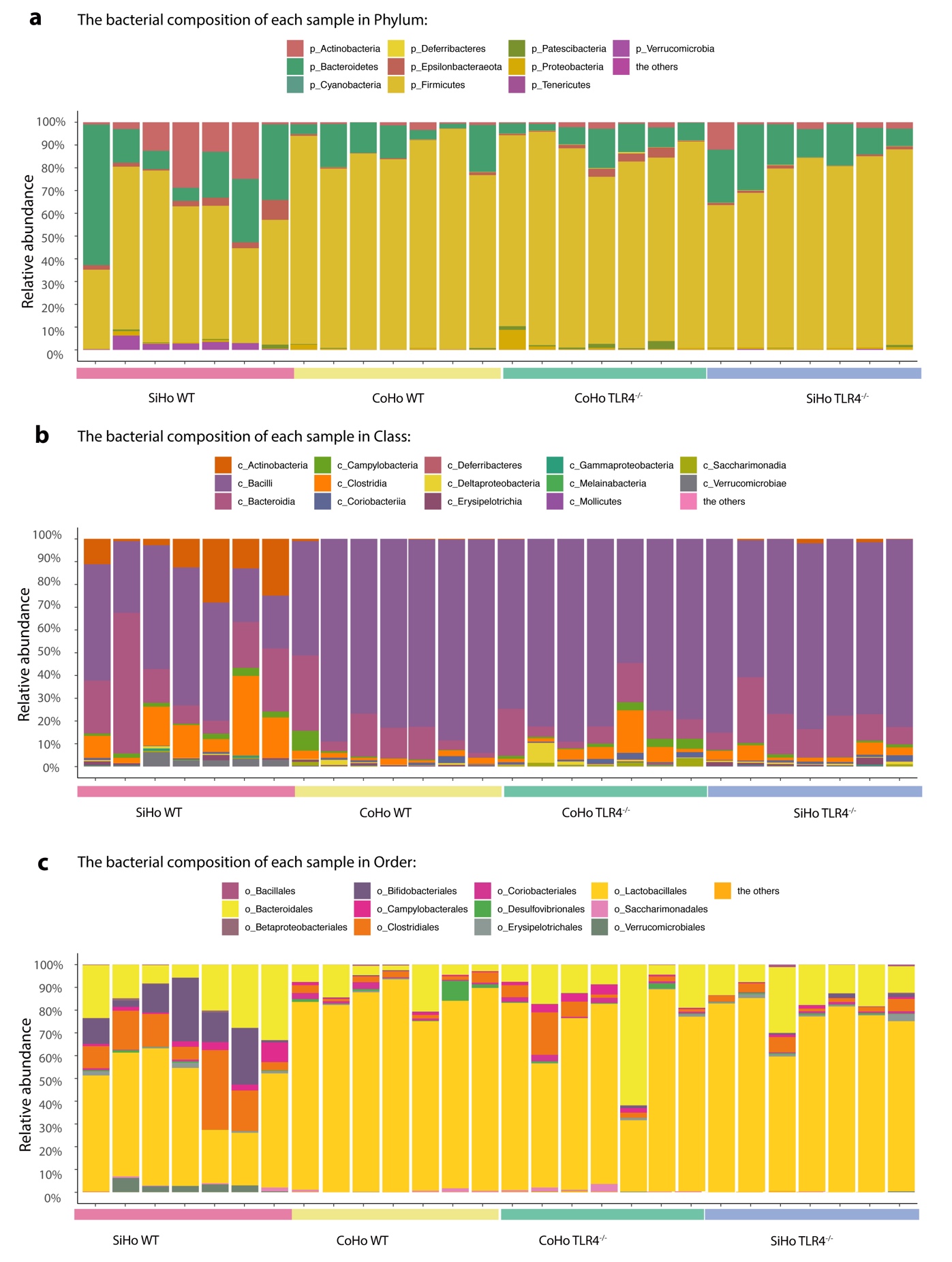
Figure S6. Gut microbiota taxonomic composition in Co-housing experiments.**

(a) Bar plots of the phylum taxonomic level. (b) Bar plots of the class taxonomic level. (c) Bar plots of the order taxonomic level. Relative abundance is plotted for each sample.

**
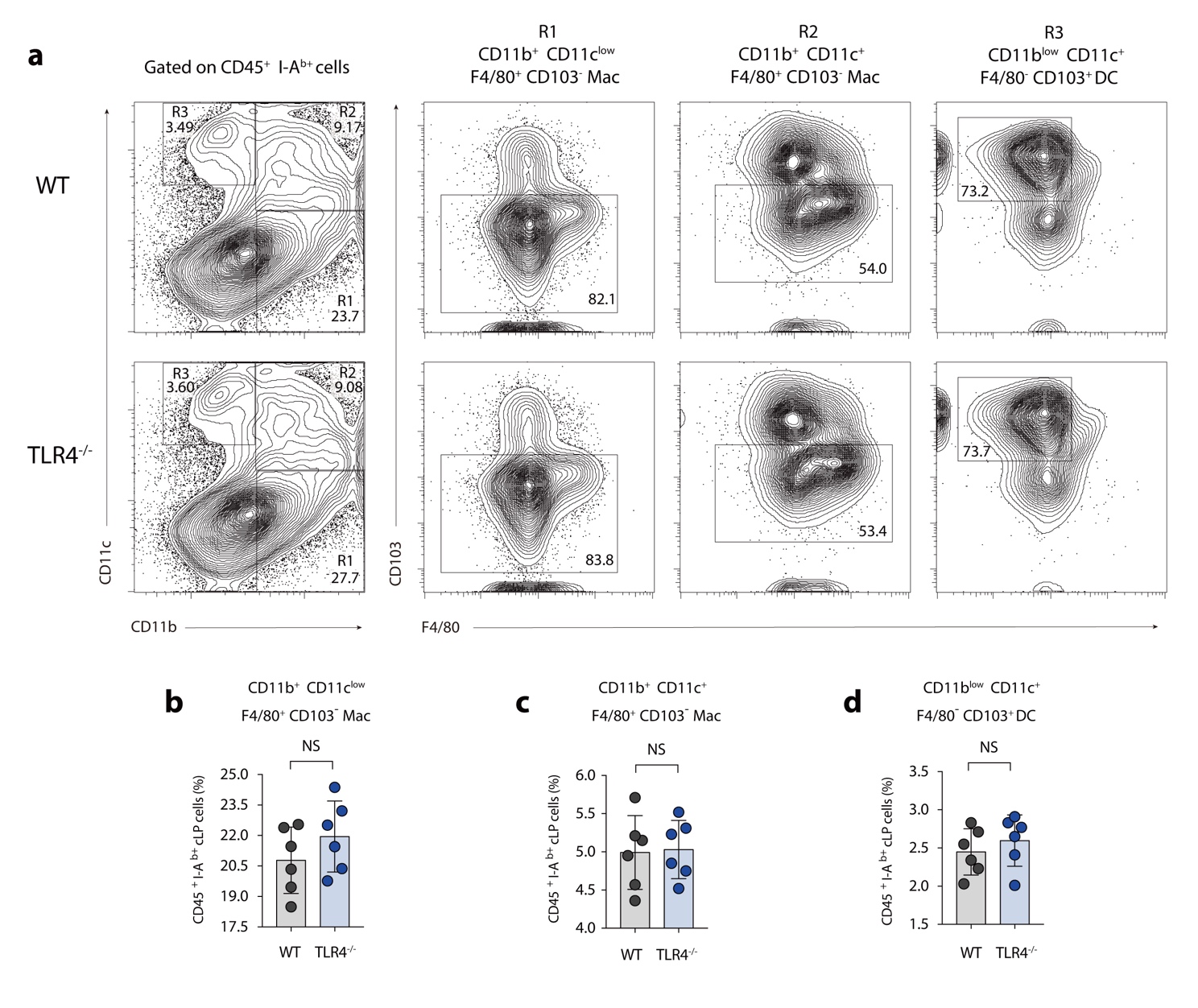
**

**Figure S7. The intestinal innate immune responses evaluation between WT and TLR4^-/-^ mice.**

(a) Representative flow cytometric analysis of colonic LP macrophage and DC subpopulations in WT and TLR4^-/-^ mice. R1 fraction, CD11b^+^ CD11c^low^ F4/80^+^ CD103^−^ macrophages; R2 fraction, CD11b^+^ CD11c^+^ F4/80^+^ CD103^−^ macrophages; R3 fraction, CD11b^low^ CD11c^+^ F4/80^−^ CD103^+^ DC. Numbers in outlined areas indicate percent cells in each gated area. (b) Statistical analysis of CD11b^+^ CD11c^low^ F4/80^+^ CD103^−^ macrophages frequency in colonic LP of WT and TLR4^-/-^ mice. (c) Statistical analysis of CD11b^+^ CD11c^+^ F4/80^+^ CD103^−^ macrophages frequency in colonic LP of WT and TLR4^-/-^ mice. (d) Statistical analysis of CD11b^low^ CD11c^+^ F4/80^−^ CD103^+^ DC frequency in colonic LP of WT and TLR4^-/-^ mice.

**
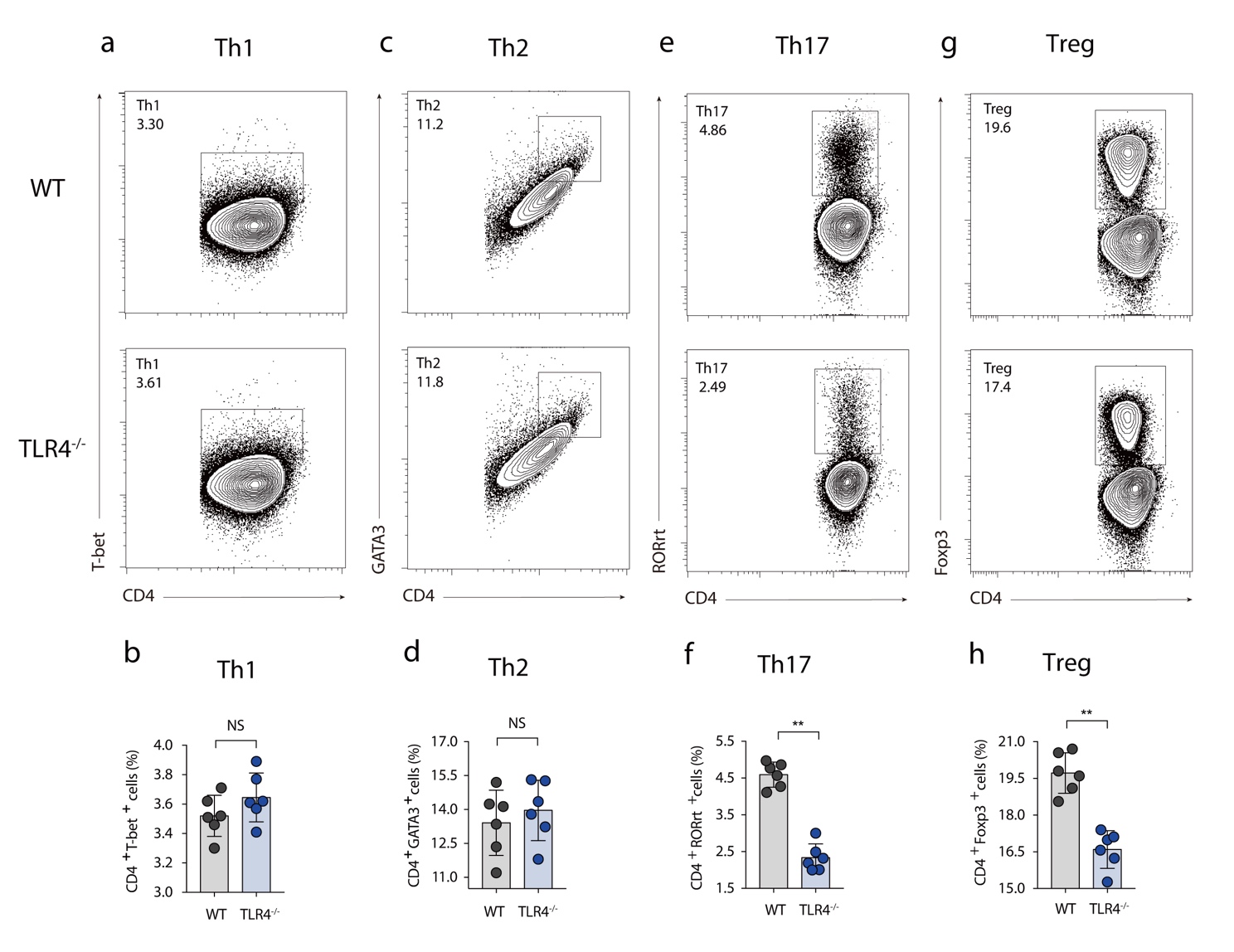
**

**Figure S8. The intestinal adaptive immune responses evaluation between WT and TLR4^-/-^ mice.**

Representative flow cytometric analysis of colonic LP CD4^+^ T cells subpopulations in WT and TLR4^-/-^ mice. (a-b) Th1 cell (co-expression of CD4 and T-bet); (c-d) Th2 cell (co-expression of CD4 and GATA3); (e-f) Th17 cell (co-expression of CD4 and RORγt); (g-h) Treg cell (co-expression of CD4 and Foxp3). Numbers in outlined areas indicate percent cells in each gated area.

**
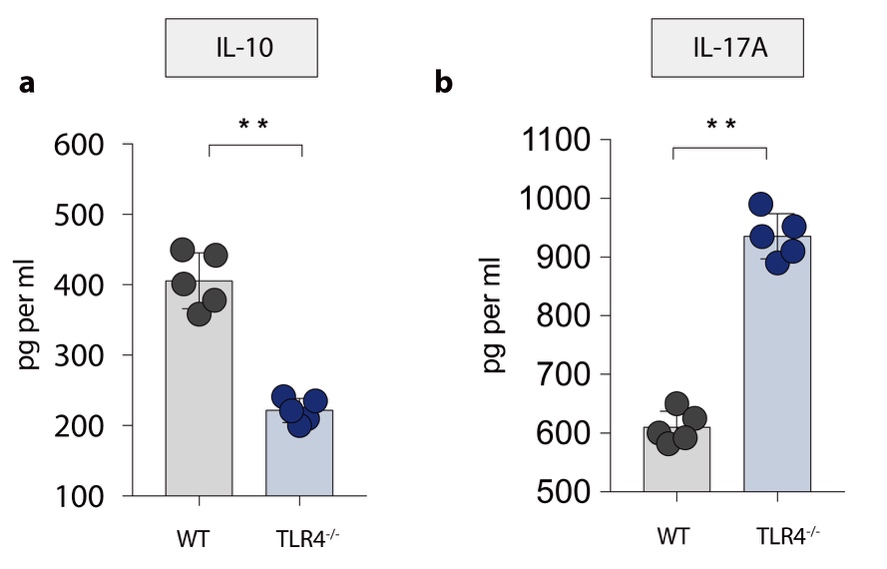
Figure S9. The cytokines profile of Treg and Th17 cells between WT and TLR4^-/-^ mice.**

(a) IL-10 cytokine level in colon tissue homogenate were measured by ELISA between WT and TLR4^-/-^ groups. (b) IL-17A cytokines levels in colon tissue homogenate were measured by ELISA between WT and TLR4^-/-^ groups.

**
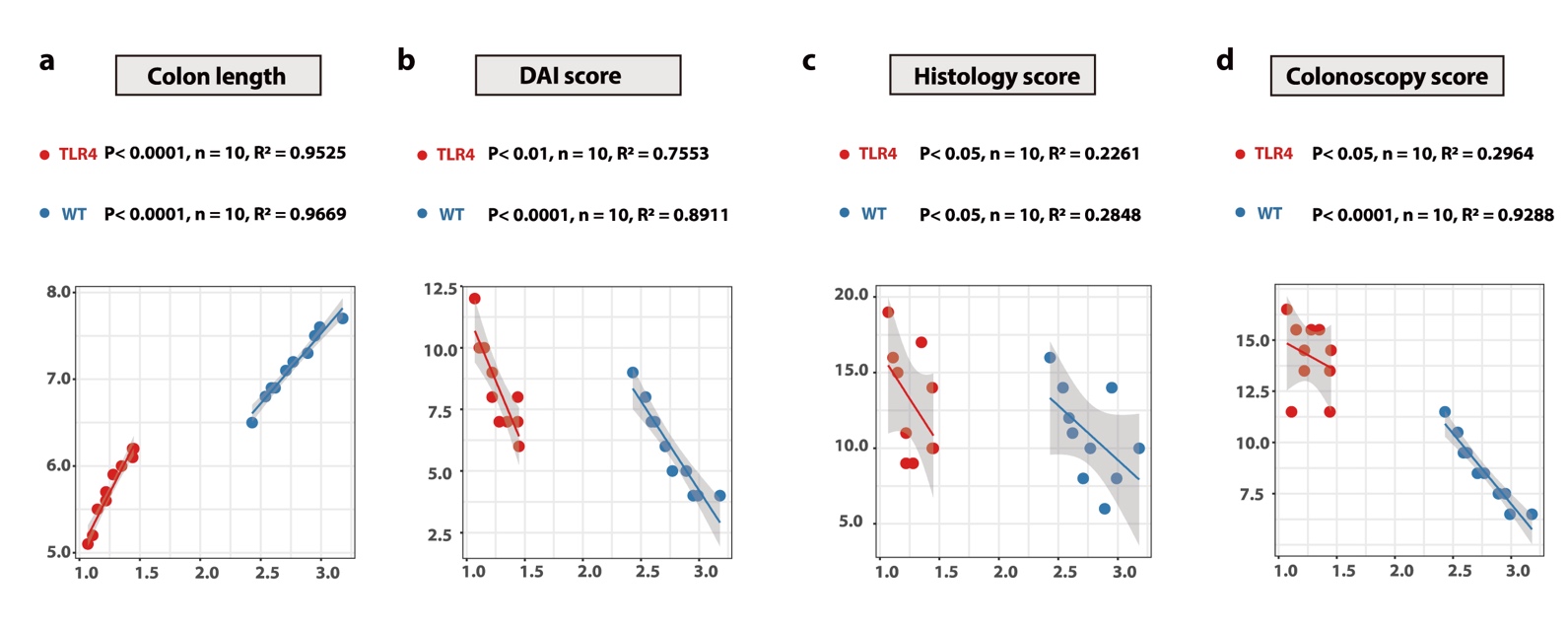
**

**Figure S10.** **Correlation analysis between RORγt^+^ Treg cells and clinical parameters.**

(a) Correlation analysis between RORγt^+^ Treg cells and colon length. (b) Correlation analysis between RORγt^+^ Treg cells and DAI score. (c) Correlation analysis between RORγt^+^ Treg cells and histology score. (d) Correlation analysis between RORγt^+^ Treg cells and colonoscopy score.

**
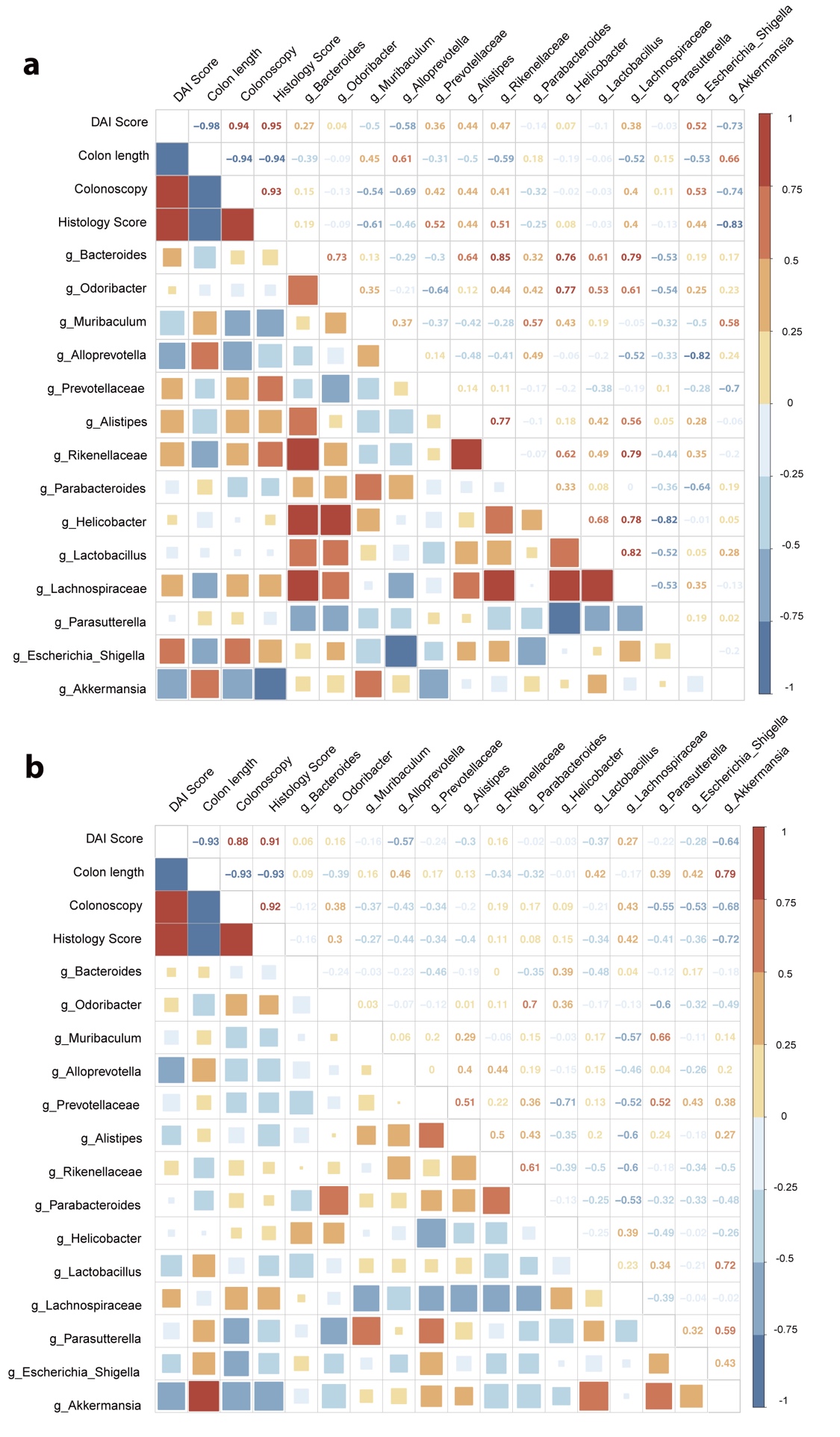
Figure S11.** **Correlation analysis between differential flora and phenotypic indicators.**

(a) Spearman Correlation Matrix between bacteria and clinical parameters in WT mice. (b) Spearman Correlation Matrix between bacteria and clinical parameters in TLR4^-/-^ mice.

**
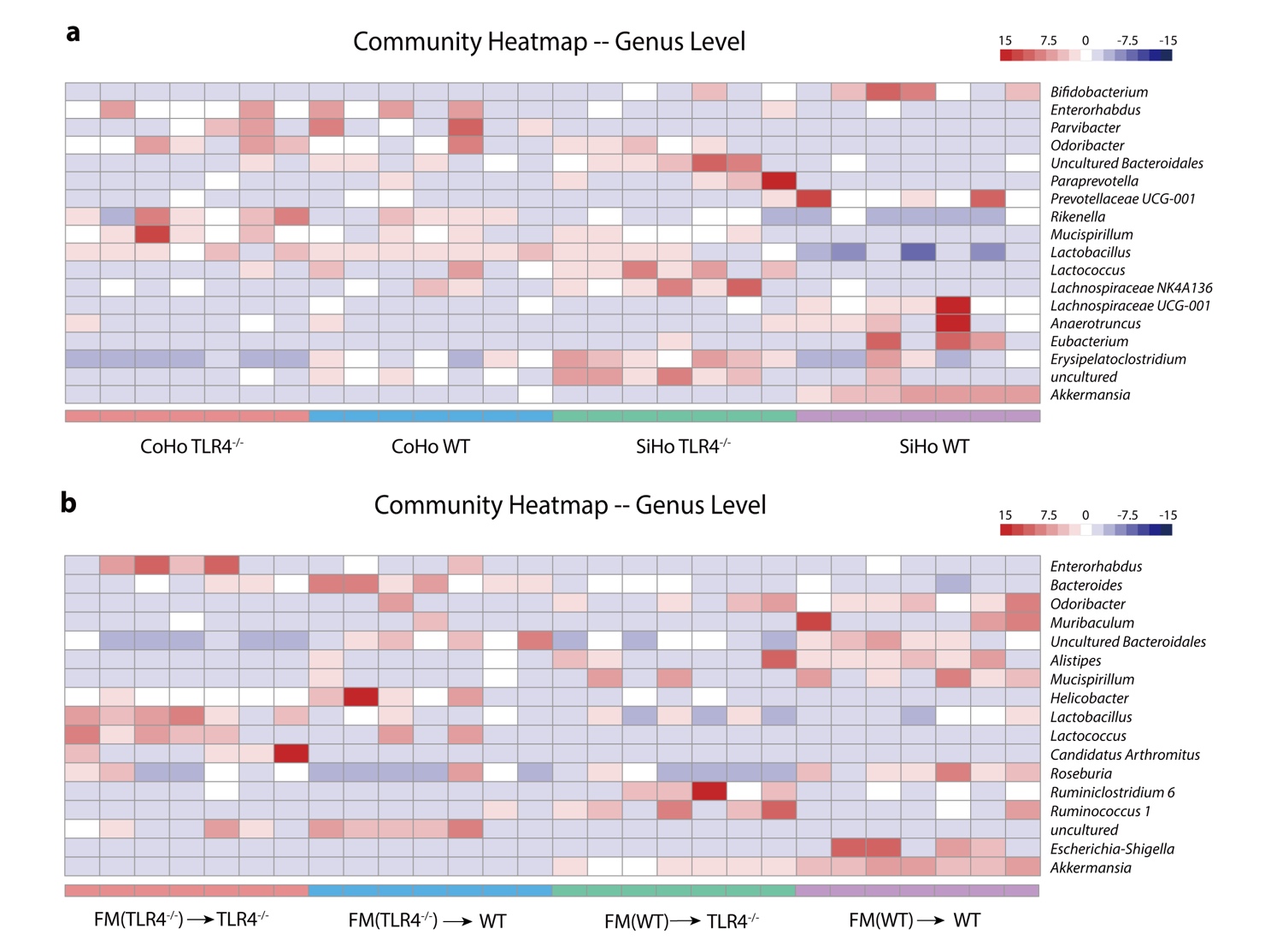
**

**Figure S12. Gut microbiota landscope of Co-housing and FMT experiments.**

(a) Heatmap of selected most differentially abundant features at the genus level among SiHo mice (SiHo WT and SiHo TLR4^-/-^) or CoHo mice (CoHo WT and CoHo TLR4^-/-^) groups.

(b) Heatmap of selected most differentially abundant features at the genus level among FMT groups.

**
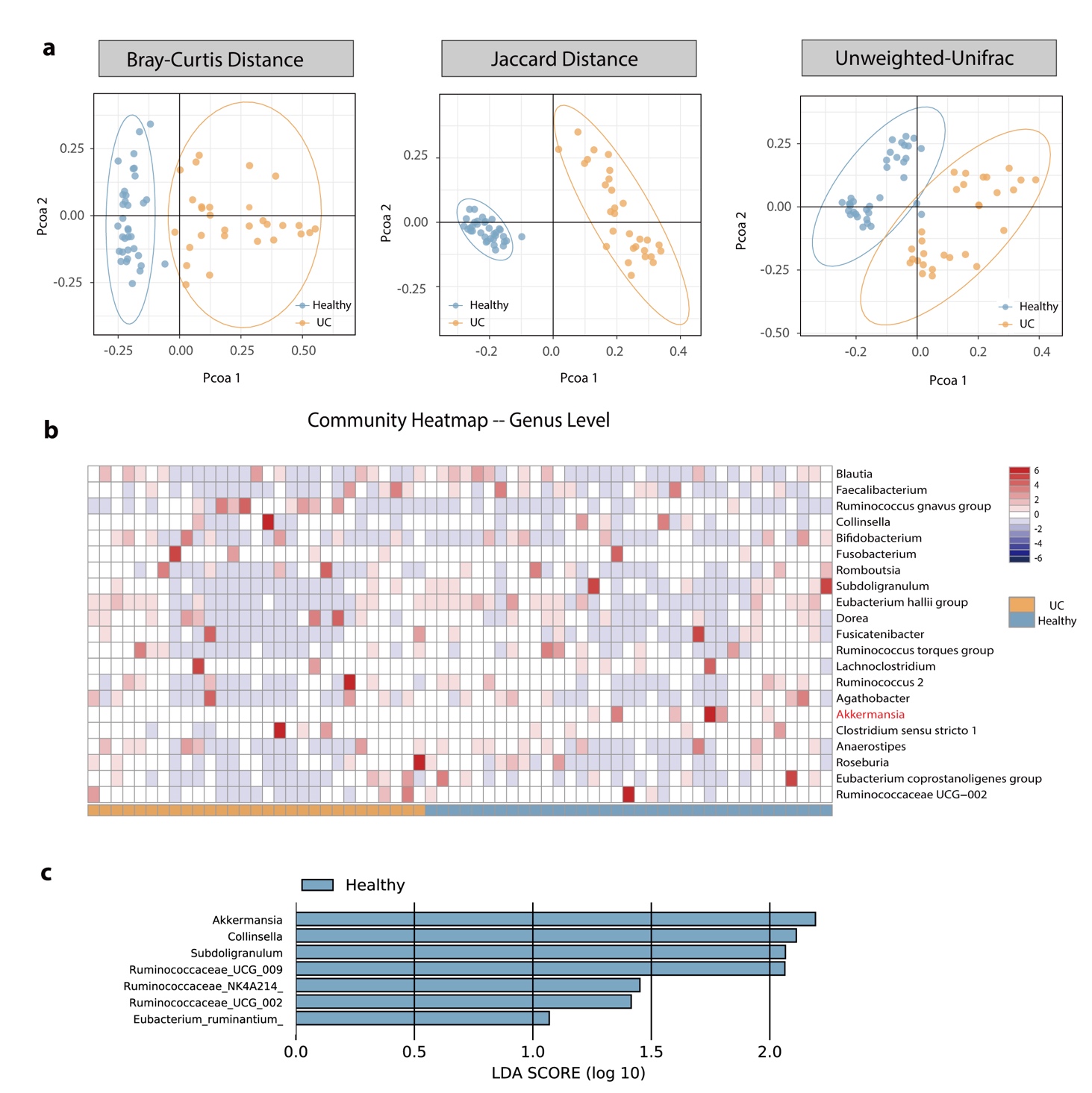
**

**Figure S13. The relative abundance of *A. muciniphila* is decreased in stool samples in patients with UC.** (a) PCoA of Beta diversity between healthy participants and patients with UC (Based on Bray-Curtis metric distances, Jaccard distances, Unweighted-UniFrac distances index). (b) Heatmap of selected most differentially abundant features at the genus level between healthy participants and patients with UC. (c) LDA score based on LEfSe analysis depicted the predominant bacteria in healthy participants. The criteria for feature selection are log LDA score > 1.0.

**
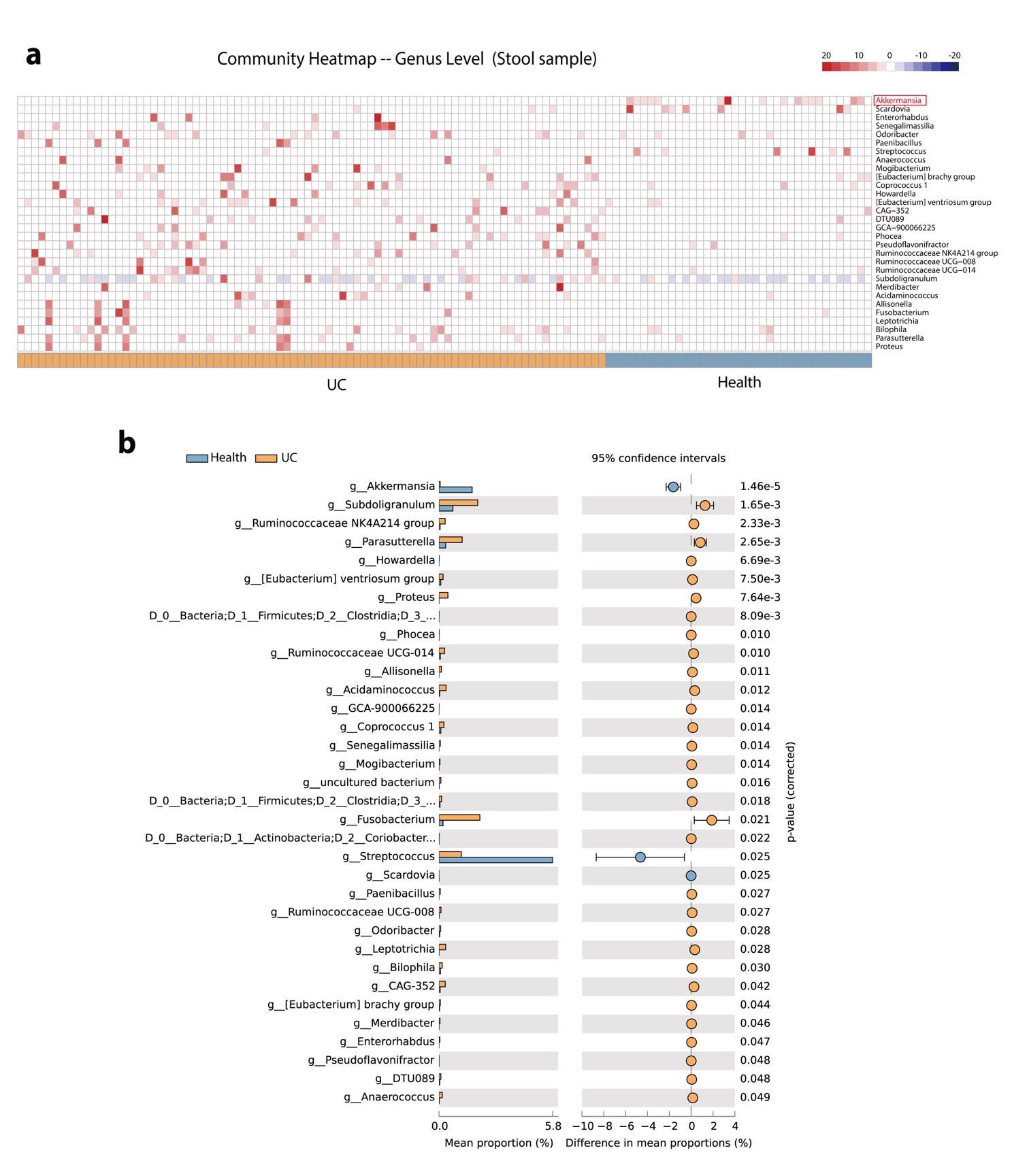
**

**Figure S14. The microbiome of UC patients are different from healthy participants.** (Based on database analysis of stool sample) (a) Comparison heatmap manifested significantly altered bacterial strains between UC patients (n = 84) with healthy participants (n = 38). Gene-microbiome-sequencing analysis of 16S rRNA on the basis of stool samples. (b) STAMP statistical analysis displayed microbiome taxonomic difference from stool samples between UC patients (n = 84) with healthy participants (n = 38).

**
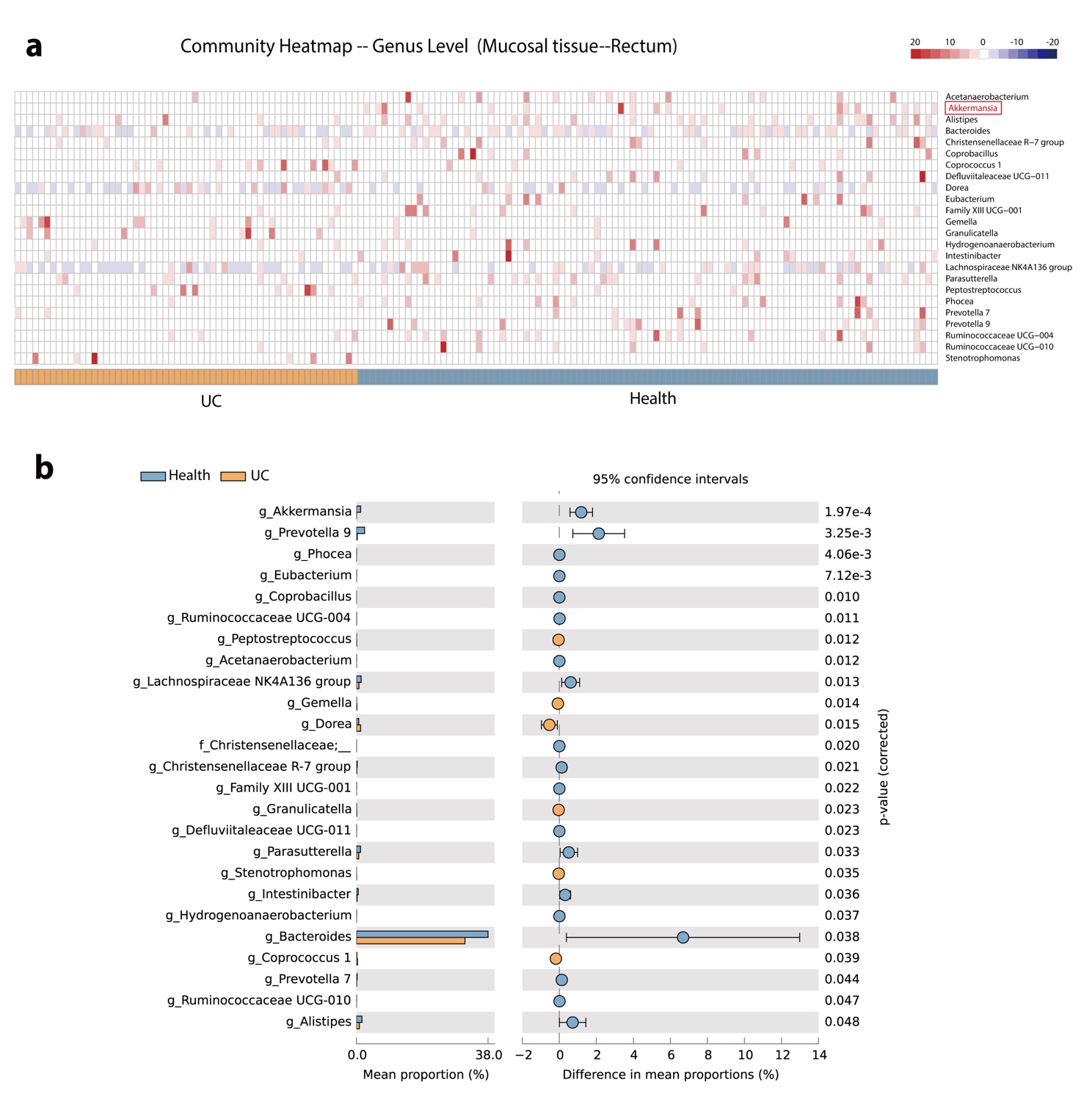
**

**Figure S15. The microbiome of UC patients are different from healthy participants.** (Based on database analysis of rectum tissue) (a) Comparison heatmap manifested significantly altered bacterial strains between UC patients (n = 58) with healthy participants (n = 98). Gene-microbiome-sequencing analysis of 16S rRNA on the basis of rectum mucosal biopsy. (b) STAMP statistical analysis displayed microbiome taxonomic difference from rectum mucosal biopsy samples between UC patients (n = 58) with healthy participants (n = 98).

**
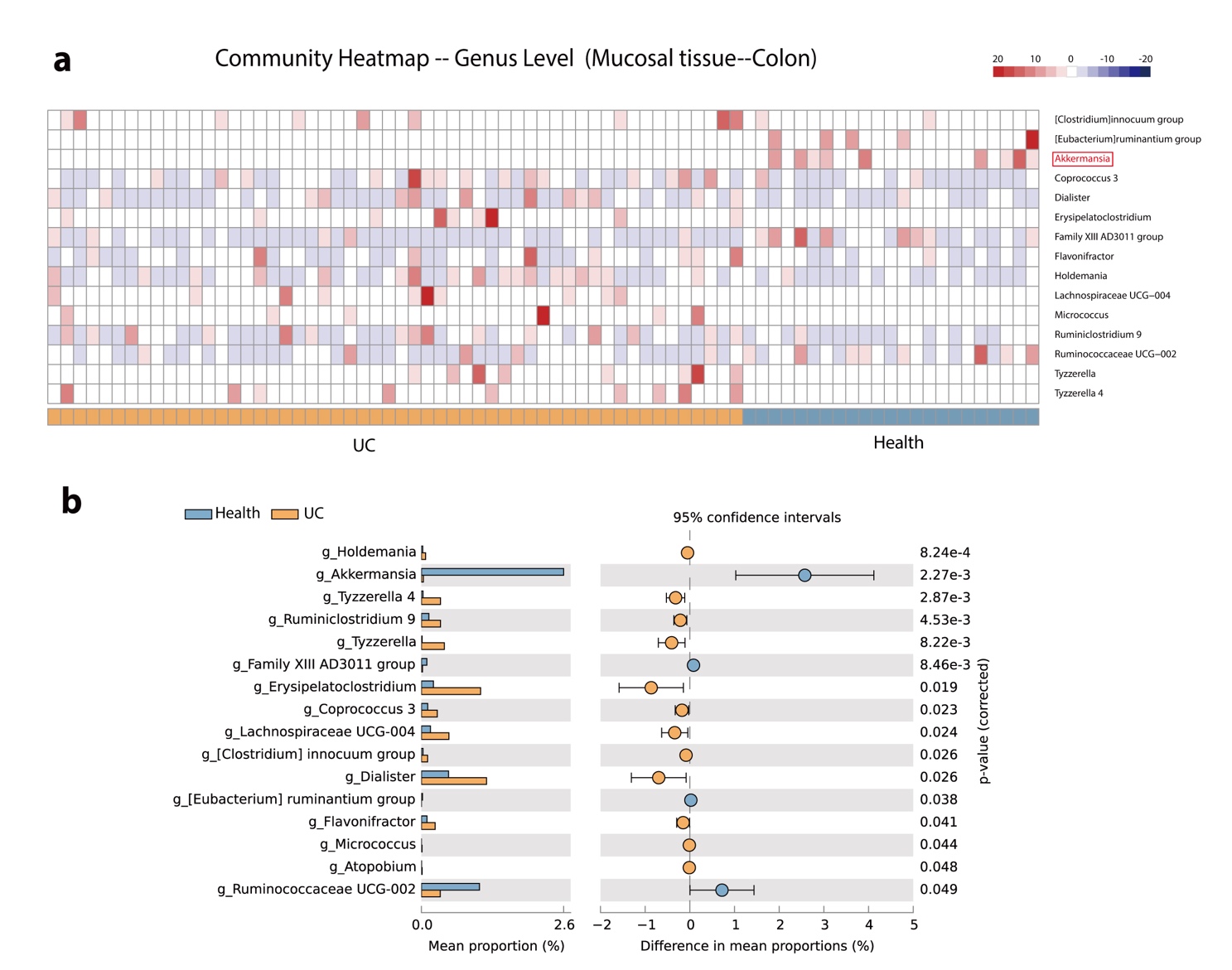
**

**Figure S16. The microbiome of UC patients are different from healthy participants.** (Based on database analysis of colon tissue) (a) Comparison heatmap manifested significantly altered bacterial strains between UC patients (n = 54) with healthy participants (n = 23). Gene-microbiome-sequencing analysis of 16S rRNA on the basis of colon mucosal biopsy. (b) STAMP statistical analysis displayed microbiome taxonomic difference from colon mucosal biopsy between UC patients (n = 54) with healthy participants (n = 23).

**
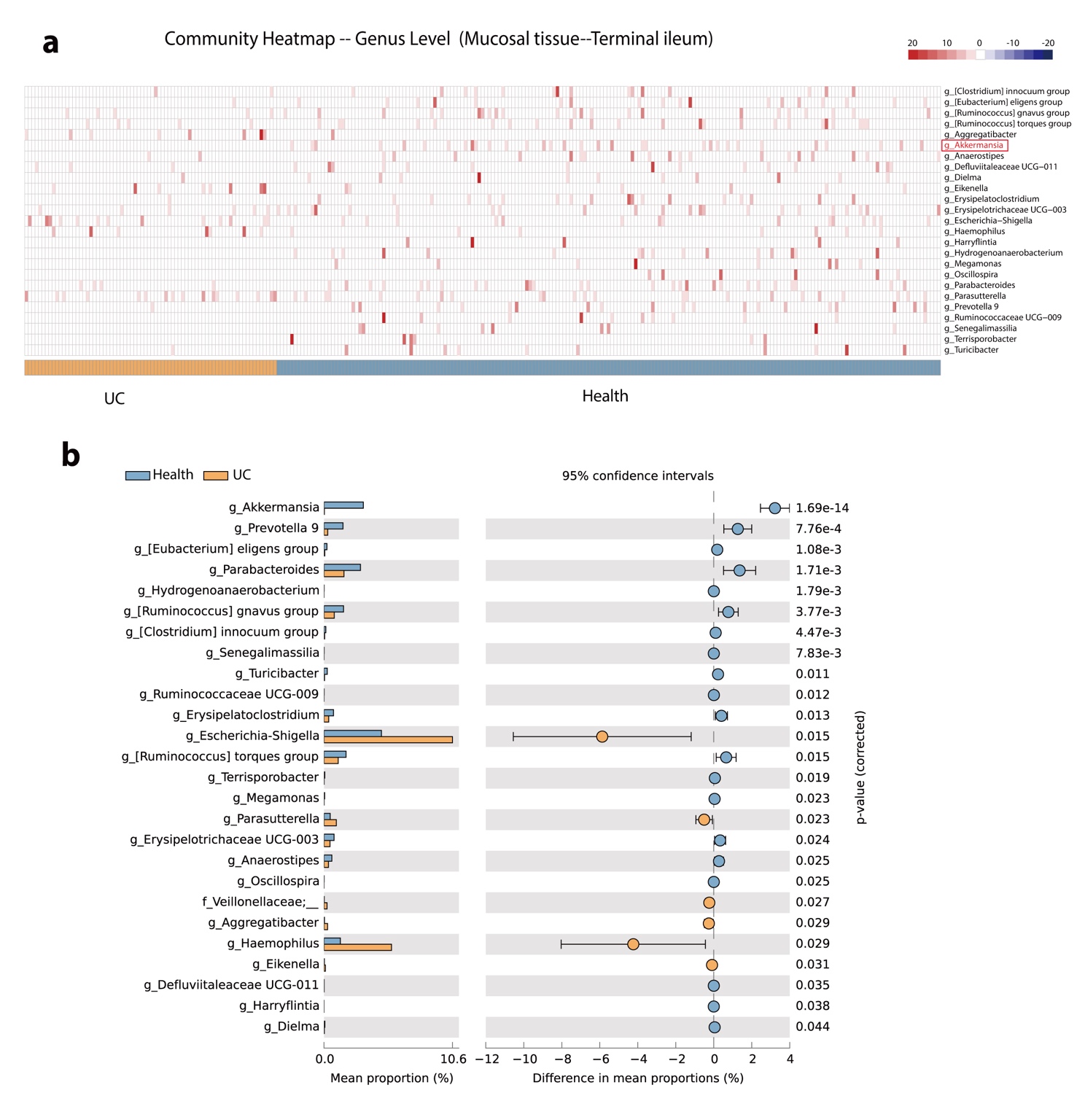
**

**Figure S17. The microbiome of UC patients are different from healthy participants.** (Based on database analysis of terminal ileum tissue) (a) Comparison heatmap manifested significantly altered bacterial strains between UC patients (n = 74) with healthy participants (n = 195). Gene-microbiome-sequencing analysis of 16S rRNA on the basis of terminal ileum mucosal biopsy. (b) STAMP statistical analysis displayed microbiome taxonomic difference from terminal ileum mucosal biopsy samples between UC patients (n = 74) with healthy participants (n = 195).

**
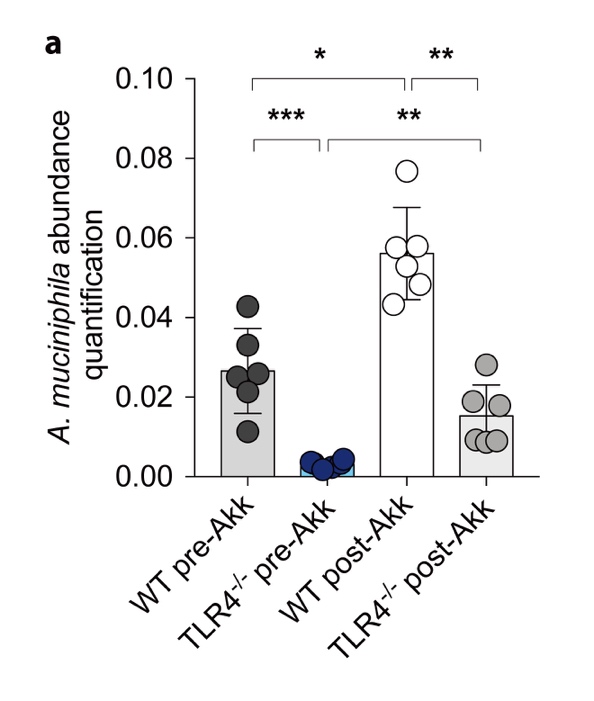
**

**Figure S18. *A. muciniphila* abundance discrepancy following single bacteria supplementation.**

Based on 16S rRNA sequencing results, *A. muciniphila* abundance among WT pre-Akk, TLR4^-/-^ pre-Akk, WT post-Akk, TLR4^-/-^ post-Akk groups were analyzed.

**
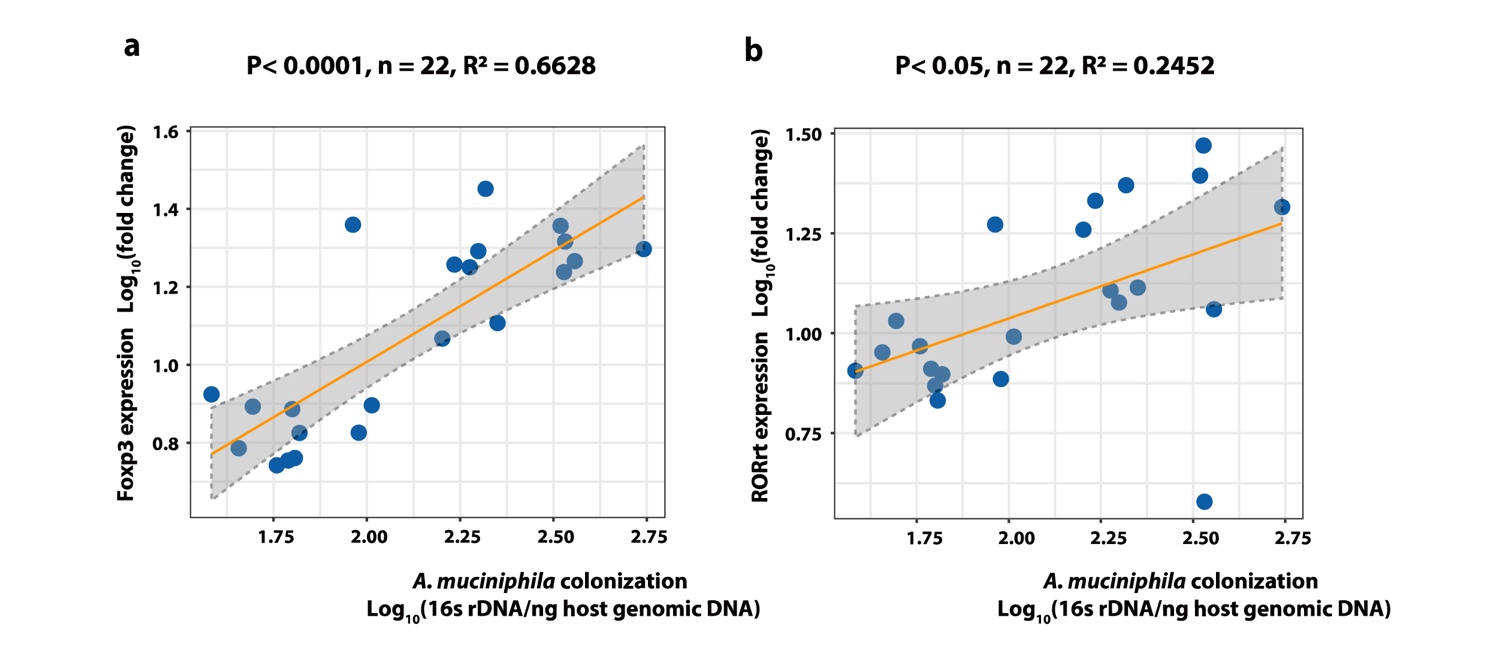
**

**Figure S19. Correlation analysis between transcription factor expression and *A. muciniphila* colonization.** (a) The correlation between Foxp3 expression and *A. muciniphila* colonization in colonic mucosa of WT mice in homeostasis was analyze. (b) The correlation between RORγt expression and *A. muciniphila* colonization in colonic mucosa of WT mice in homeostasis was analyze.

**
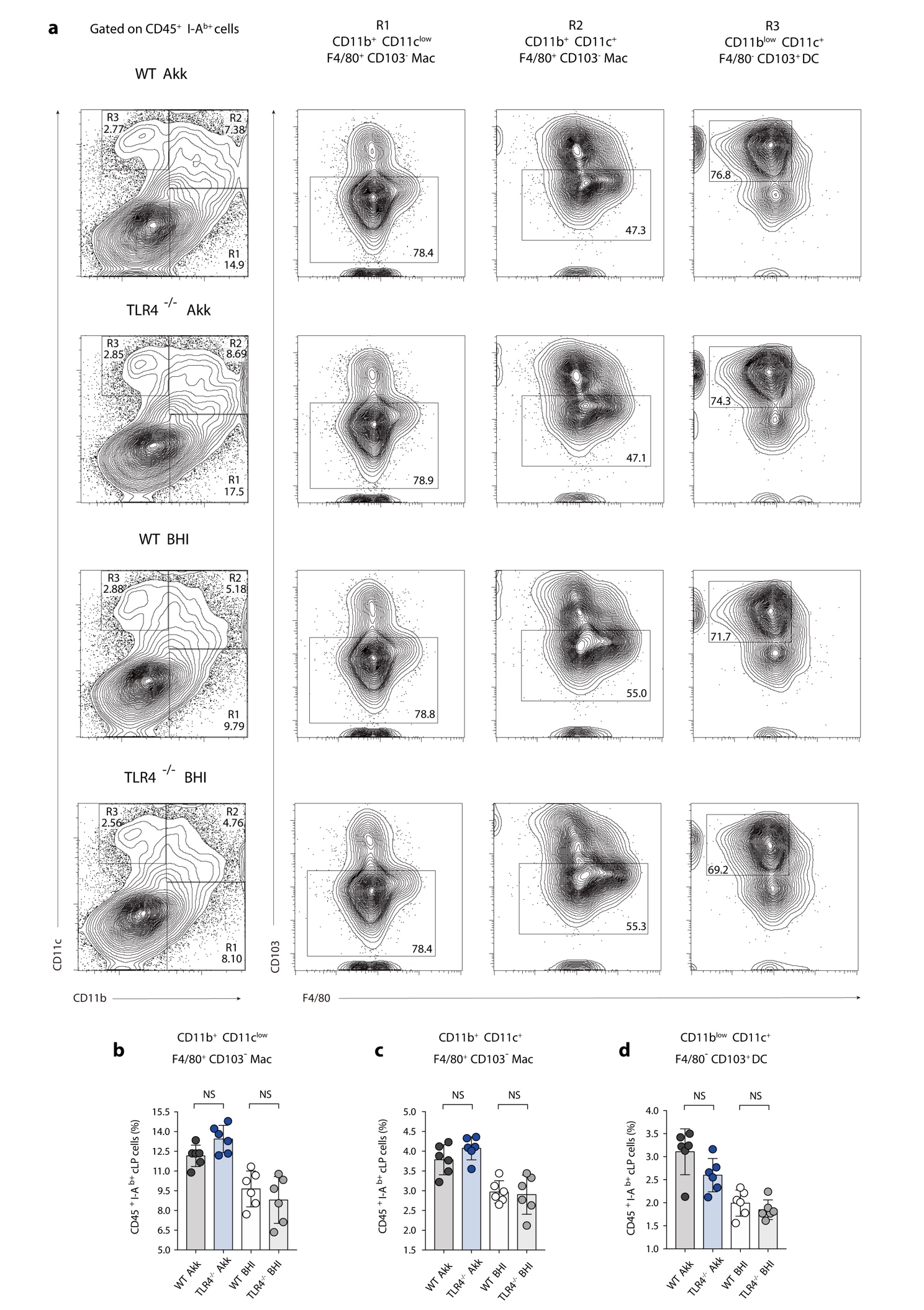
**

**Figure S20. The intestinal innate immune responses evaluation between WT and TLR4^-/-^ mice after *A. muciniphila* supplementation.** (a) Representative flow cytometric analysis of colonic LP macrophage and DC subpopulations among WT Akk, TLR4^-/-^ Akk, WT BHI, TLR4^-/-^ BHI groups. (b) Statistical analysis of CD11b^+^ CD11c^low^ F4/80^+^ CD103^−^ macrophages. (c) Statistical analysis of CD11b^+^ CD11c^+^ F4/80^+^ CD103^−^ macrophages. (d) Statistical analysis of CD11b^low^ CD11c^+^ F4/80^−^ CD103^+^ DC.

**
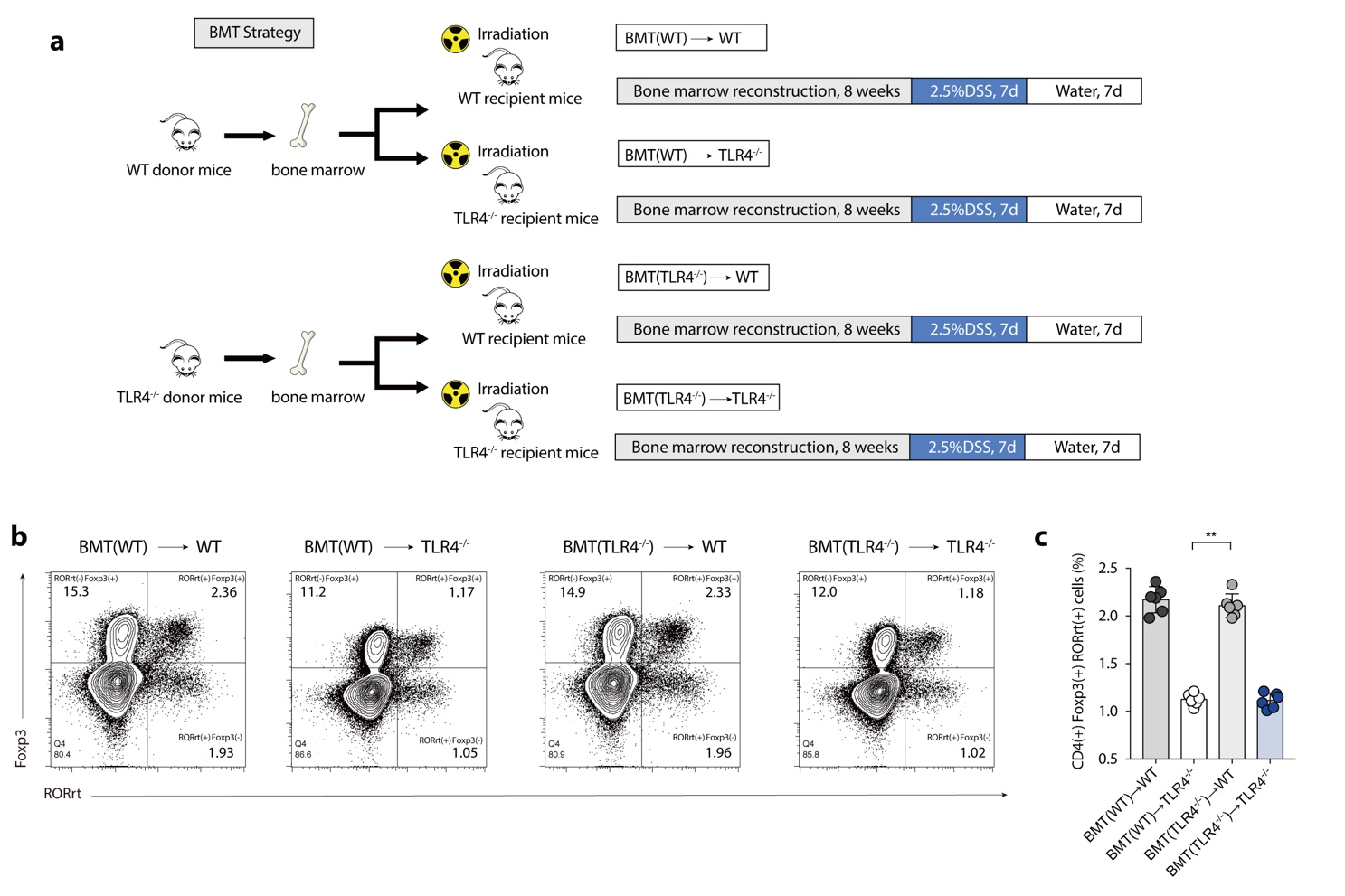
**

**Figure S21. Intestinal epithelial-derived TLR4 pathway participating in intestinal immune activation against colitis.** (a) Bone-marrow transplantation (BMT) strategy. (b) Representative flow cytometric analysis of colonic RORγt^+^ Treg cells among BMT groups. (c) Statistical analysis of CD4 ^(+)^ Foxp3 ^(+)^ RORγt ^(+)^ T cells frequency in BMT groups.


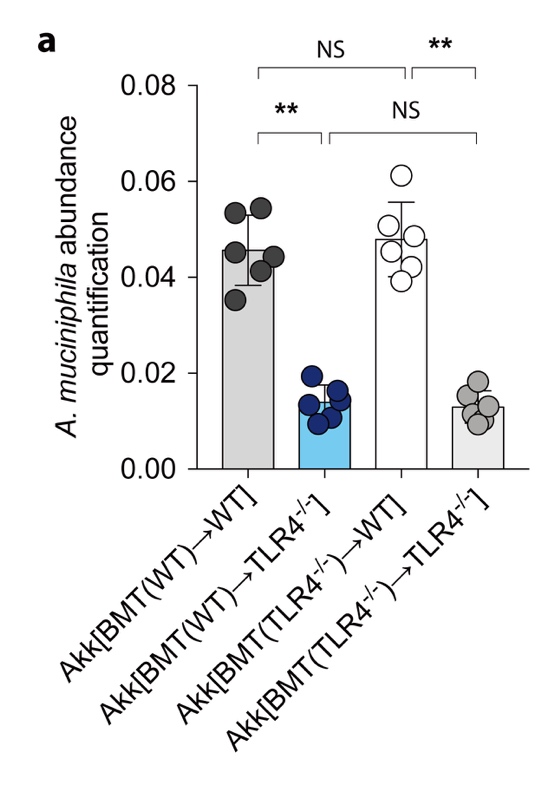


**Figure S22. *A. muciniphila* abundance discrepancy in BMT experiment following bacteria supplementation.** Based on 16S rRNA sequencing results, *A. muciniphila* abundance among Akk[BMT(WT)→TLR4^-/-^], Akk[BMT(TLR4^-/-^)→WT], Akk[BMT(WT)→WT], Akk[BMT(TLR4^-/-^)→TLR4^-/-^] groups were analyzed.

**
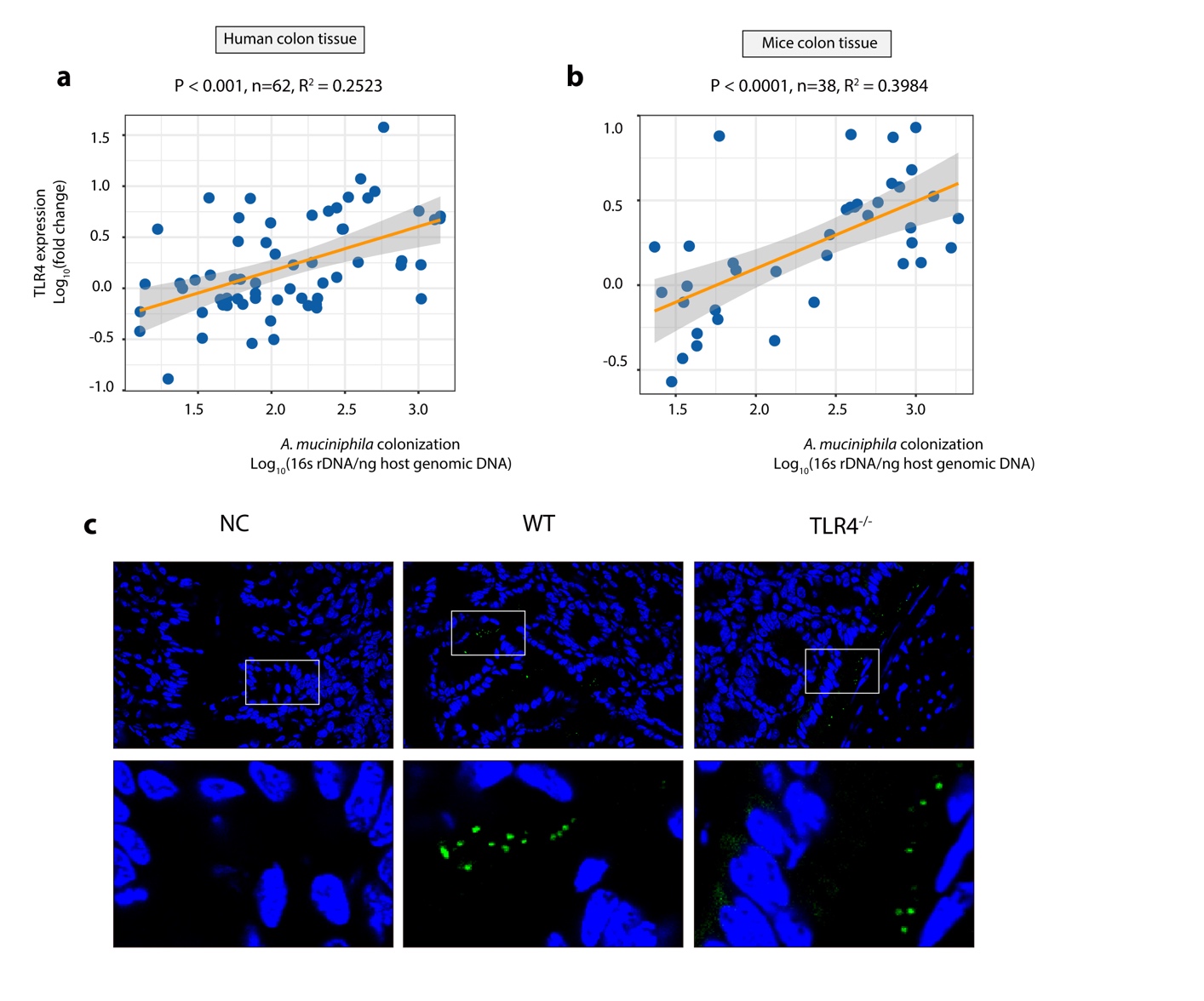
**

**Figure S23. TLR4 affects the intestinal colonization of *A. muciniphila* during homeostasis**

(a) The correlation between TLR4 expression and *A. muciniphila* colonization in colonic mucosa of healthy participants in homeostasis was analyze. (b) The correlation between TLR4 expression and *A. muciniphila* colonization in colonic mucosa of WT mice in homeostasis was analyze. (c) Fluorescence in situ hybridization (FISH) shows *A. muciniphila* (MUC1437) in the colonic epithelium and lumen in WT and TLR4^-/-^ mice. The lower panel shows a higher magnification of insert shown in the uppper panel (white rectangle). Epithelial nuclei are stained with DAPI (Blue).


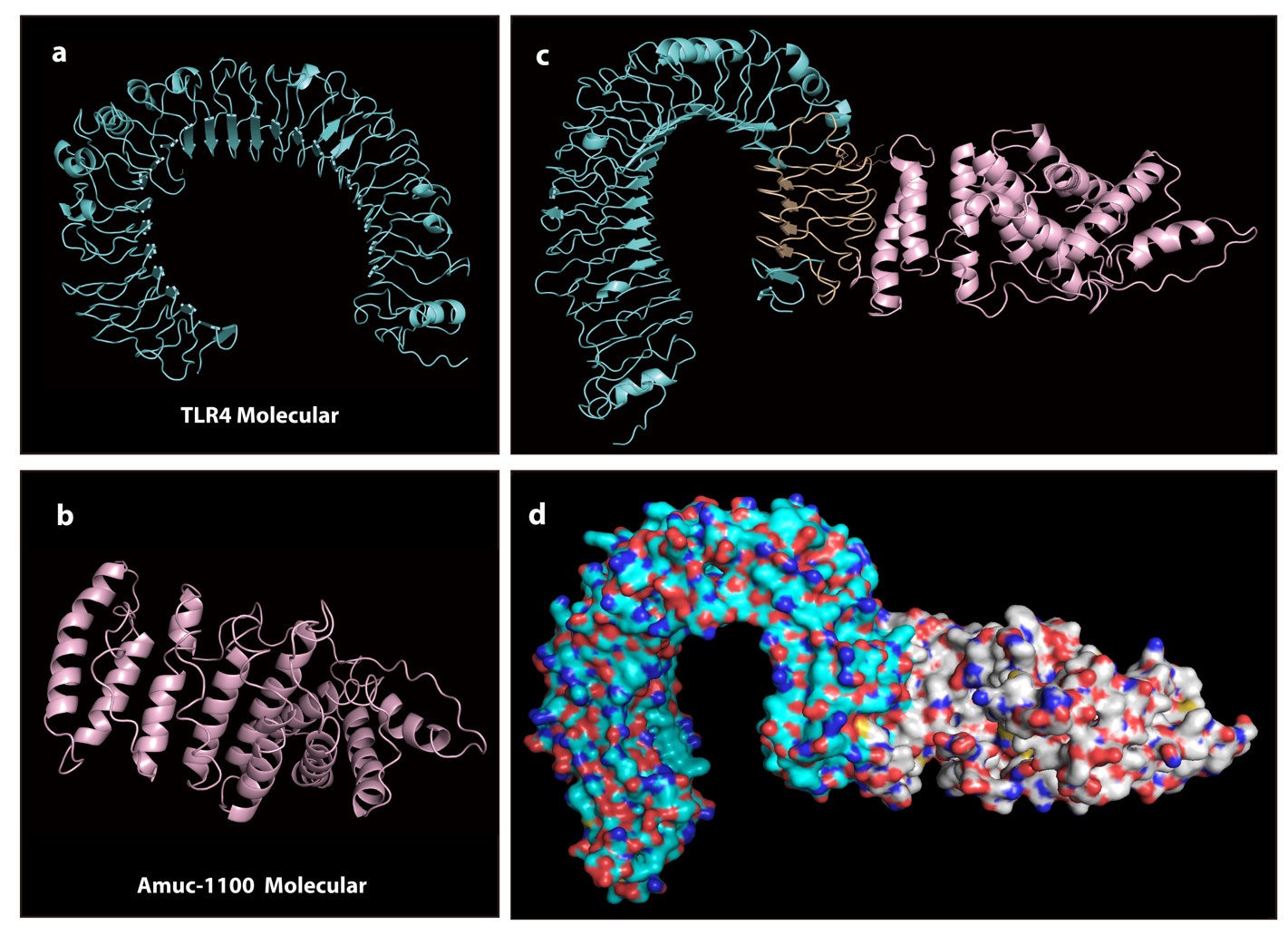
**Figure S24. The Interaction between TLR4 and *Amuc-1100* mediated the intestinal colonization of *A. muciniphila***

(a) TLR4 molecular structure. (b) *Amuc-1100* molecular structure constructed. (<https://zhanglab.ccmb.med.umich.edu/I-TASSER/>). (c) Top-scoring Z-dock prediction (medium accuracy) was displayed by native chain structure. Orange color part manifested the interface of TLR4 and *Amuc-1100.* (d) Interface of the top-scoring Z-dock prediction superimposed on the three-dimensional crystal complex.


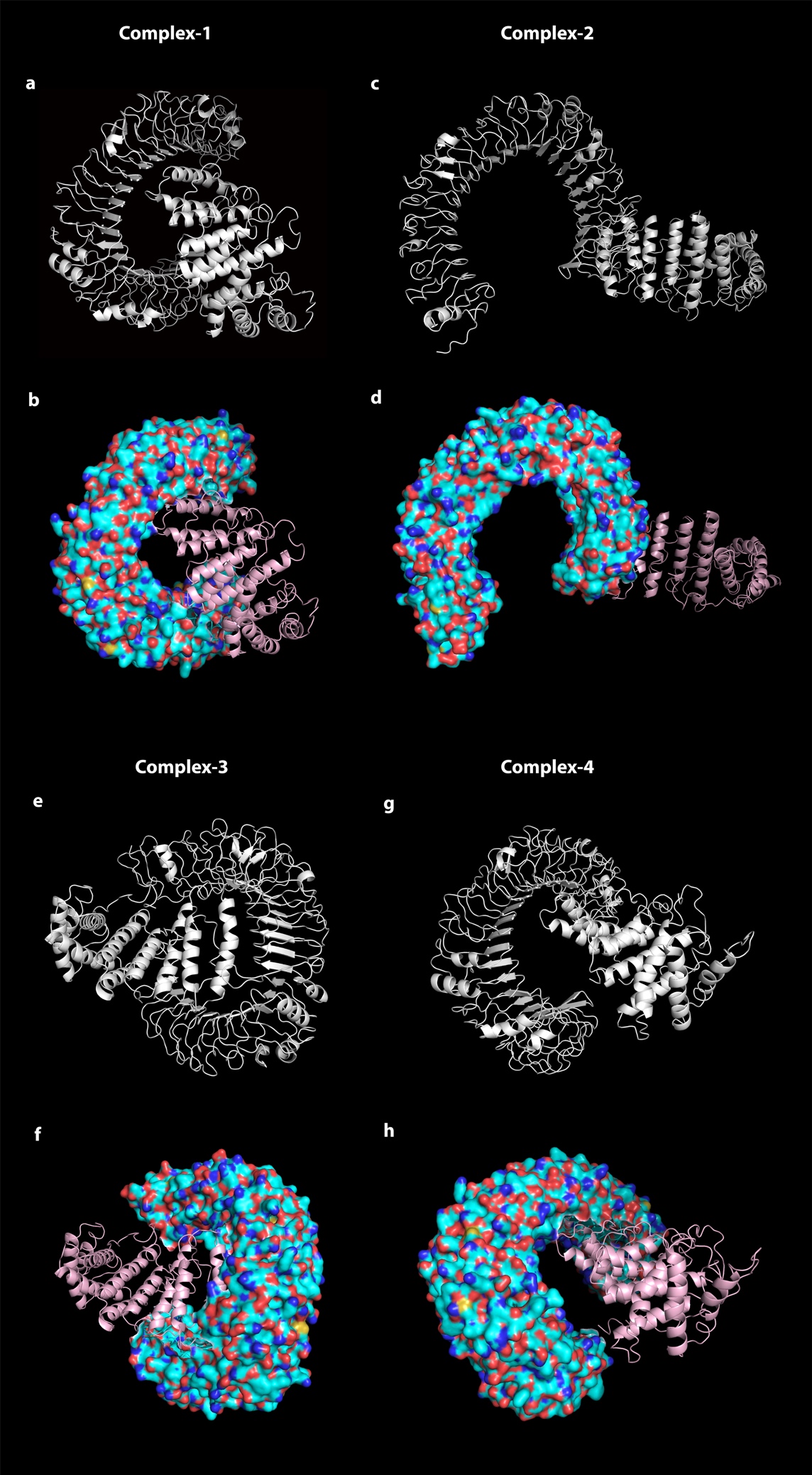


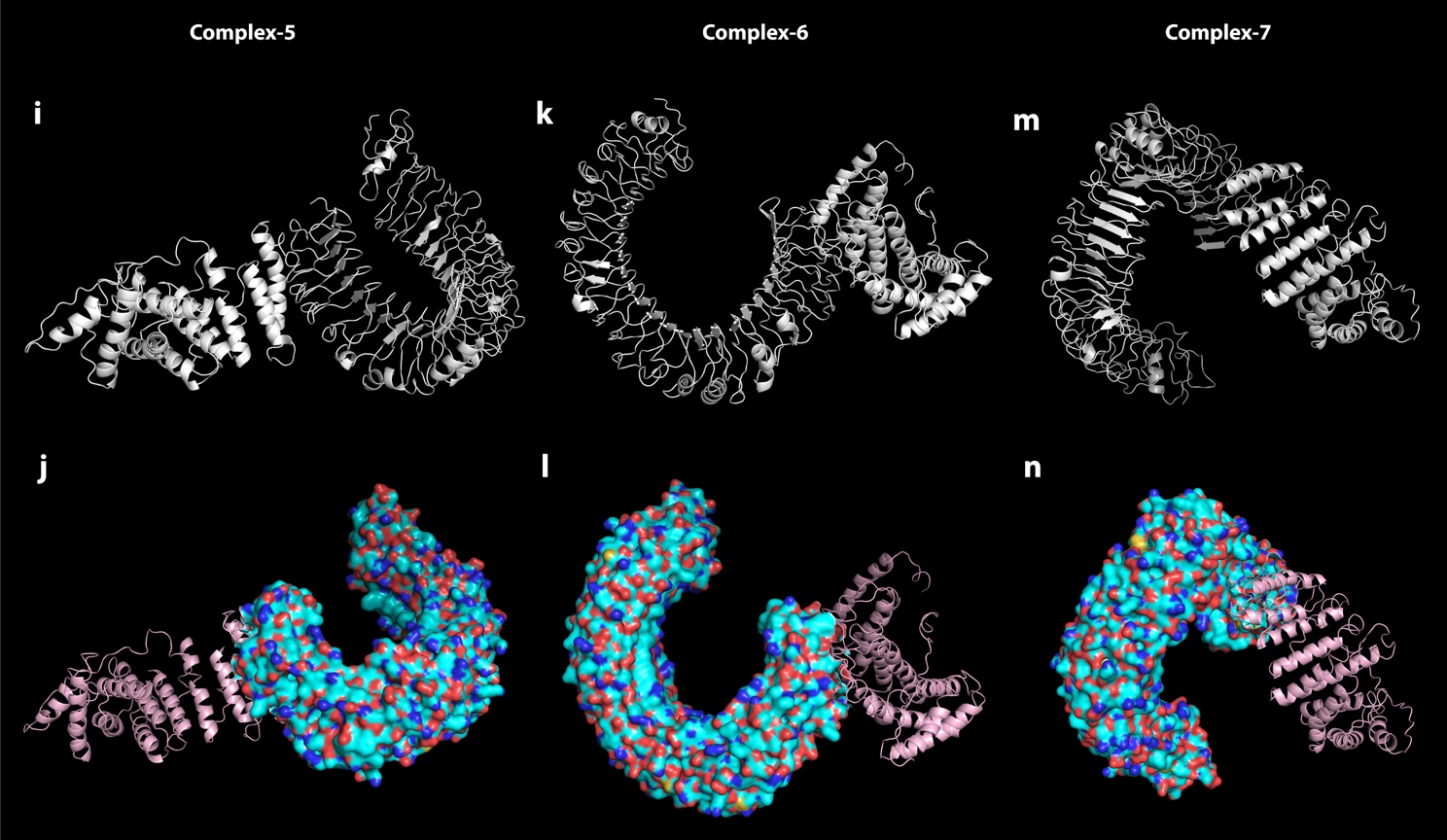


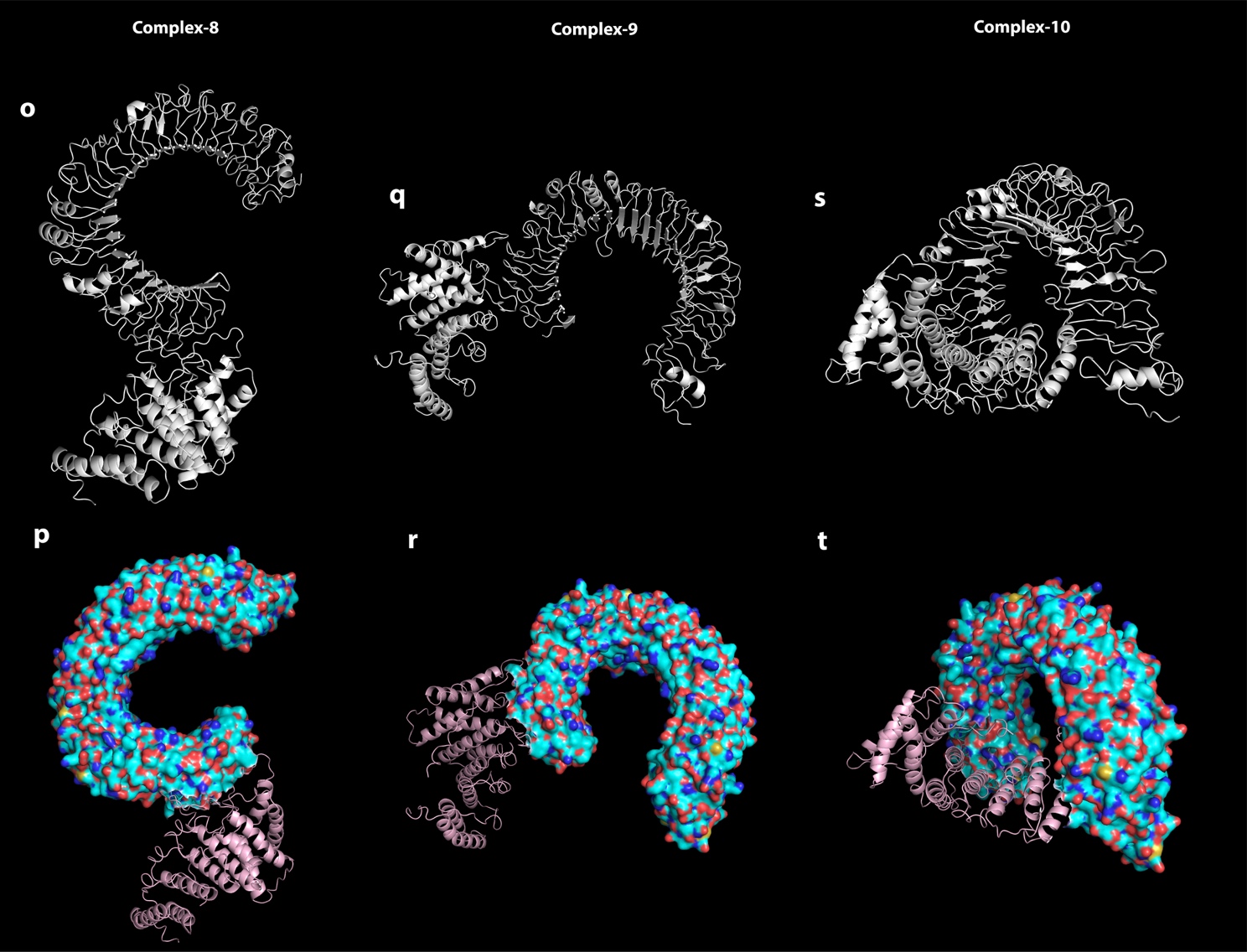


**Figure S25-S27. Top-ranked possible complex scenarios of TLR4 and *Amuc-1100* based on ZDOCK prediction.**

(a) Z-dock prediction of native configuration 1. (b) Complex 1 superimposed on the three-dimensional crystal complex. (c) Z-dock prediction of native configuration 2. (d) Complex 2 superimposed on the three-dimensional crystal complex. (e) Z-dock prediction of native configuration 3. (f) Complex 3 superimposed on the three-dimensional crystal complex. (g) Z-dock prediction of native configuration 4. (h) Complex 4 superimposed on the three-dimensional crystal complex. (i) Z-dock prediction of native configuration 5. (j) Complex 5 superimposed on the three-dimensional crystal complex. (k) Z-dock prediction of native configuration 6. (l) Complex 6 superimposed on the three-dimensional crystal complex. (m) Z-dock prediction of native configuration 7. (n) Complex 7 superimposed on the three-dimensional crystal complex. (o) Z-dock prediction of native configuration 8. (p) Complex 8 superimposed on the three-dimensional crystal complex. (q) Z-dock prediction of native configuration 9. (r) Complex 9 superimposed on the three-dimensional crystal complex. (s) Z-dock prediction of native configuration 10. (t) Complex 10 superimposed on the three-dimensional crystal complex.

**supplemental Methods**

**Faecal genomic DNA extraction and 16S-rRNA sequencing**

Stool samples were freshly collected from live mice or subjects, snap-frozen and stored at -80 °C. DNA was extracted from the stool using the Power Faecal^®^ DNA Isolation Kit (MoBio Carlsbad, CA USA) according to the manufacturers’ instructions. The specific 341F: CCTAYGGGRBGCASCAG, 806R: GGACTACNNGGGTATCTAAT primers were used to amplify the V3-V4 region of the 16S rRNA gene. A GeneJET Gel Extraction Kit (Thermo Fisher Scientific, Cat. No. K0691, Waltham, U.S.A.) was used for PCR product purification. The libraries were sequenced by Novogene Co., Ltd (Tianjin, China) using the Illumina NovaSeq 6000 platform. The sequencing data were processed using the Quantitative Insights into Microbial Ecology 2 (QIIME2, version 2020. 2 [1]) toolkit in a 64 CPU threads 768 GB RAM Ubuntu 20.04 server. First, V3-V4 primers were trimmed by cutadapt 3.1[2]. Then, the trimmed sequences were imported into the QIIME2 pipeline using the 'qiime tools import' command. Imported sequences were denoised into amplicon sequence variants (ASVs) using the 'qiime dada2 denoise-paired' command. The output of this command included the representative sequence of each ASV and the feature table of each sample. As DADA2 [3] contains internal chimaera checking methods and abundance filtering, no additional filtering processes were needed. The 'qiime feature-table summarize' command was used to generate a summary report of the feature table. Samples with less than 8000 nonchimeric sequences (sampling depth) were excluded from further analysis. All samples were rarefied to the same depth by using the 'qiime feature-table rarefy' command, and a rarefied feature table was the output. Alpha-diversity and beta-diversity were calculated via the 'qiime diversity alpha' and 'qiime diversity beta' commands from the rarefied feature table, respectively. The principal coordinate analysis (PCoA) results were calculated and visualized by the 'qiime diversity pcoa' and 'qiime emperor plot' commands. The significance of the dissimilarity of each sample was calculated by analysis of similarities (ANOSIM). A pretrained naive Bayes classifier (silva-132-99-nb-classifier.qza, trained on SILVA [4] 132 99% full-length sequences) and the 'qiime feature-classifier classify-sklearn' command were used to explore the taxonomic composition of the samples. The abundance of bacteria between groups was ranked according to the linear discriminant analysis (LDA) effect size (LEfSe) (http://huttenhower.sph.harvard.edu/lefse) [5].

**Antibiotic cocktail treatment**

Eight-week-old male WT or TLR4^-/-^ mice harbouring a conventional microbiota were transferred to sterile cages and received broad-spectrum antibiotic cocktails (vancomycin, 100 mg/kg; neomycin sulphate 200 mg/kg; metronidazole 200 mg/kg; and ampicillin 200 mg/kg) intragastrically once a day for 5 days for gut microbiota depletion [6]. To control the microbiological status of mice, a fresh antibiotic mixture was mixed at every feeding point, and individual faecal samples were collected following 5 days of antibiotic protocols. Cultural and 16S PCR amplification methods revealed pseudo germ-free conditions in antibiotic-treated mice as previously reported [7].

**Co-housing experiment**

For the cohousing experiments, 3- to 4-week-old male WT or TLR4^-/-^ littermates originating from the same breeders (heterozygote TLR4^+/-^ breeder pairs) were divided to be either housed singly (SiHo WT, Cage No. 1; SiHo TLR4^-/-^, Cage No. 2) or cohoused with age- and sex-matched mice (CoHo WT and CoHo TLR4^-/-^ mice at a 1:1 ratio) (Cage No. 3) for 6 weeks [8, 9]. Stool samples were harvested, and faecal DNA was sequenced after 6 weeks of cohousing. CoHo mice were compared with their SiHo littermates as controls. Subsequently, both SiHo mice and CoHo mice were administered 2.5% (w/v) DSS in their drinking water ad libitum for 7 days followed by 7 days of normal water for colitis evaluation.

**Faecal microbiota transplantation (FMT)**

For FMT experiments, the homogenate of frozen faeces from WT or TLR4^-/-^ donor (8-week-old) mice was prepared in sterile PBS under anaerobic conditions and subsequently filtered through a sterile 70-μm strainer to remove large faecal particles. The filtrate was centrifuged at 3000 × g for 10 min and resuspended in normal saline for transplantation, as previously described [10]. For better intestinal flora colonization, recipient mice underwent 5 days of antibiotic cocktail treatments for gut microbiota depletion before FMT. Subsequently, each antibiotic-treated mouse was administered 150 μL of faecal suspension containing 1×10^10^ bacteria via oral gavage once a day for 7 days. To ensure the intestinal colonization status of FMT procedures, mice were then allowed to rest for 3 days, and faecal samples were collected before DSS administration.

**Single-cell isolation**
Single-cell suspensions were generated from the spleen and the colonic lamina propria (LP) as described previously [11]. In brief, spleens were smashed over 70-μm cell strainers before red cell lysis using ACK buffer (1.5 M NH4Cl; 100 mM KHCO3, 10 mM EDTA). For isolation of LP lymphocytes, colonic tissues were splayed longitudinally with the removal of mucus by scraping and then stored in complete RPMI with 10% foetal bovine serum (FBS). Supernatants were discarded after filtering through 100-μm cell strainers, and the remaining tissue was incubated in HBSS (without Ca^2+^ and Mg^2+^) containing 5 mM EDTA and 1 mM DL-dithiothreitol (DTT) for 45 min at 37 °C on a shaker (250 rpm). Supernatants were filtered through 70-μm cell strainers and discarded, and the remaining tissue was incubated for 45 min at 37 °C on a shaker (250 rpm) in digestion solution (HBSS containing 5% FCS, 2.5 mg/ml collagenase IV, 4 mg/ml dispase, 0.2 mg/ml DNase I). Cells were passed through 70-μm strainers, washed with PBS, and used for FACS analysis.

**Flow cytometry**

Cells were phenotypically analysed by multicolour flow cytometry (Coulter Cytomics FC 500, Beckman Coulter, Fullerton, USA) using Coulter Epics Expo 32 software. For extracellular surface staining, cells were preincubated with mouse Fcγ II/III CD16/CD32 Receptors Blocking Reagent (BioLegend, Clone 93, Cat. No. 101320, Dilution 1:1000) for 15 min at 4 °C and were then stained with the following labelled antibodies for 30 min on ice: PerCP-conjugated anti-CD45 (BioLegend, Clone 30-F11, Cat. No. 103130, Dilution 1:200), PE-CD103 (BioLegend, Clone 2E7, Cat. No. 121406, dilution 1:100), PE/Cyanine7-conjugated anti-CD11b (BioLegend, M1/70, Cat. No. 1010216, dilution 1:100), APC-conjugated anti-MHCII (BioLegend, Clone M5/114.15.2, Cat. No. 107613, dilution 1:100), Brilliant Violet 421-conjugated anti-F4/80 (BioLegend, Clone BM8, Cat. No. 123131, dilution 1:100), Brilliant Violet 510-conjugated anti-CD11c (BioLegend, Clone N418, Cat. No. 117337, Dilution 1:100). For T cell phenotyping associated with intracellular cytokine staining, cells were originally stained with the following surface staining antibodies: APC-Cy7-conjugated anti-live/dead dye (Invitrogen, Cat. No. L34975, Dilution 1:200), BV510-conjugated anti-CD4, (BioLegend, Clone RM4-5, Cat. No. 100559, dilution 1:200), PerCP-conjugated anti-CD45, (BioLegend, Clone 30-F11, Cat. No. 103130, Dilution 1:200). Cells were harvested, washed in FACS buffer (PBS, 2% FCS), fixed and permeabilized with Fixation/Permeabilization working solution (Invitrogen, Cat. No. 005523) and permeabilization buffer for 20 min at RT in the dark. Cells were washed and resuspended in permeabilization buffer before the addition of intracellular cytokine antibodies: PE-conjugated anti-RORγt (BD, Clone Q31-378, Cat. No. 562607, dilution 1:100), FITC-conjugated anti-T-bet (BioLegend, Clone 4B10, Cat. No. 644812, dilution 1:100), AF647-conjugated Foxp3 (BioLegend, Clone 150D, Cat. No. 320014, Dilution 1:100), BV421-conjugated GATA3 (BioLegend, Clone 16E10A23, Cat. No. 653814, Dilution 1:100). Dead cells and doublets were excluded from the mononuclear cell population based on appropriate forward- and side-scatter plots. Appropriate isotype control fluorochrome-conjugated antibodies were used for the gate settings.

**Gut microbiota qPCR quantification**

Faecal genomic DNA was obtained as previously described. Metagenomic DNA from intestine tissue was extracted using a MINI-tissue sample genomic DNA extraction kit (Cat. No. DP316, TIANGEN Biotech, Beijing, China) according to the manufacturer’s instructions. The primers and probes used to detect the different bacteria were based on 16S rRNA gene sequences: Total Bacteria (Bacteria Universal) F-ACTCCTACGGGAGGCAGCAG, R-ATTACCGCGGCTGCTGG; *Akkermansia muciniphila* F-CAGCACGTGAAGGTGGGGAC, *Akkermansia muciniphila* R-CCTTGCGGTTGGCTTCAGAT [12]. Detection was achieved with a STEPONE PLUS instrument and software (Applied Biosystems, Foster City, CA, USA) using MESA FAST qPCR MasterMix Plus for the SYBR Assay (Eurogentec, Verviers, Belgium). Each assay was performed in duplicate in the same run. The cycle threshold of each sample was then compared to a standard curve (performed in triplicate) that was generated by diluting genomic DNA (fivefold serial dilution) (BCCM/LMG, Ghent, Belgium and DSMZ, Braunshweig, Germany).

**Cultivation of *A. muciniphila* and Mouse Colonization with *A. muciniphila***

*A. muciniphila* MucT (ATCC BAA835) was cultured under strictly anaerobic conditions at 37 °C in brain heart infusion (BHI) medium as described previously [13, 14], and exponentially growing cultures were washed with PBS and immediately frozen in PBS containing 25% glycerol to a final concentration of 1×10^10^ cells per mL [15]. Prior to administration, a frozen pellet of *A. muciniphila* was thawed and resuspended in anaerobic PBS to a concentration of 1.5×10^9^ per mL. Mice were treated by oral gavage with a bacterial *A. muciniphila* suspension in BHI twice a week for 3 weeks. BHI broth was used as a vehicle control. After *A. muciniphila* or BHI supplementation, the mice were administered DSS 1 week after the final gavage.

**Meta-analysis of microbiome changes in patients with UC**

For microbiome changes in patients with IBD, we downloaded the published raw 16S rRNA gene sequencing file from the open-source microbiome deposition site QIITA (https://qiita.ucsd.edu/) under study ID 1939. The raw data for the 16S microbiome sequencing results from healthy control subjects and patients with UC from the file were reanalysed and compared. Stool samples from 38 healthy control subjects and 84 UC patients were analysed, and significantly altered bacterial strains were identified between the two groups. According to the different intestinal locations of the lesions, tissue biopsy samples (rectum region: 98 healthy participants and 58 UC patients; colon region: 23 healthy participants and 54 UC patients; terminal ileum: 195 healthy participants and 74 UC patients) were also compared.

1 Bolyen E, Rideout JR, Dillon MR, Bokulich NA, Abnet CC, Al-Ghalith GA*, et al.* Reproducible, interactive, scalable and extensible microbiome data science using QIIME 2. Nature biotechnology 2019;**37**:852-7.

2 Martin M. Cutadapt removes adapter sequences from high-throughput sequencing reads. EMBnet journal 2011;**17**:10-2.

3 Callahan BJ, McMurdie PJ, Rosen MJ, Han AW, Johnson AJA, Holmes SP. DADA2: high-resolution sample inference from Illumina amplicon data. Nature methods 2016;**13**:581-3.

4 Quast C, Pruesse E, Yilmaz P, Gerken J, Schweer T, Yarza P*, et al.* The SILVA ribosomal RNA gene database project: improved data processing and web-based tools. Nucleic acids research 2012;**41**:D590-D6.

5 Segata N, Izard J, Waldron L, Gevers D, Miropolsky L, Garrett WS*, et al.* Metagenomic biomarker discovery and explanation. Genome biology 2011;**12**:1-18.

6 Heimesaat MM, Bereswill S, Fischer A, Fuchs D, Struck D, Niebergall J*, et al.* Gram-negative bacteria aggravate murine small intestinal Th1-type immunopathology following oral infection with Toxoplasma gondii. J Immunol 2006;**177**:8785-95.

7 Radulovic K, Manta C, Rossini V, Holzmann K, Kestler HA, Wegenka UM*, et al.* CD69 regulates type I IFN-induced tolerogenic signals to mucosal CD4 T cells that attenuate their colitogenic potential. J Immunol 2012;**188**:2001-13.

8 Chen L, Wilson JE, Koenigsknecht MJ, Chou WC, Montgomery SA, Truax AD*, et al.* NLRP12 attenuates colon inflammation by maintaining colonic microbial diversity and promoting protective commensal bacterial growth. Nat Immunol 2017;**18**:541-51.

9 Lei Y, Tang L, Liu S, Hu S, Wu L, Liu Y*, et al.* Parabacteroides produces acetate to alleviate heparanase-exacerbated acute pancreatitis through reducing neutrophil infiltration. Microbiome 2021;**9**:115.

10 Sun J, Xu J, Ling Y, Wang F, Gong T, Yang C*, et al.* Fecal microbiota transplantation alleviated Alzheimer's disease-like pathogenesis in APP/PS1 transgenic mice. Transl Psychiatry 2019;**9**:189.

11 Spalinger MR, Schmidt TS, Schwarzfischer M, Hering L, Atrott K, Lang S*, et al.* Protein tyrosine phosphatase non-receptor type 22 modulates colitis in a microbiota-dependent manner. J Clin Invest 2019;**129**:2527-41.

12 Schneeberger M, Everard A, Gomez-Valades AG, Matamoros S, Ramirez S, Delzenne NM*, et al.* Akkermansia muciniphila inversely correlates with the onset of inflammation, altered adipose tissue metabolism and metabolic disorders during obesity in mice. Sci Rep 2015;**5**:16643.

13 Derrien M, Van Baarlen P, Hooiveld G, Norin E, Muller M, de Vos WM. Modulation of Mucosal Immune Response, Tolerance, and Proliferation in Mice Colonized by the Mucin-Degrader Akkermansia muciniphila. Front Microbiol 2011;**2**:166.

14 Hanninen A, Toivonen R, Poysti S, Belzer C, Plovier H, Ouwerkerk JP*, et al.* Akkermansia muciniphila induces gut microbiota remodelling and controls islet autoimmunity in NOD mice. Gut 2018;**67**:1445-53.

15 Grander C, Adolph TE, Wieser V, Lowe P, Wrzosek L, Gyongyosi B*, et al.* Recovery of ethanol-induced Akkermansia muciniphila depletion ameliorates alcoholic liver disease. Gut 2018;**67**:891-901.
